# Prompting Interfacial Elastic Instability for Effective and Durable Icephobic Surfaces

**DOI:** 10.1002/advs.202503855

**Published:** 2025-06-10

**Authors:** Xinyu Fan, Jingtong Li, Deyu Yang, Jie Fei, Hejun Li, Xianghui Hou

**Affiliations:** ^1^ State Key Laboratory of Solidification Processing Shaanxi Key Laboratory of Fiber Reinforced Light Composite Materials Northwestern Polytechnical University Xi'an 710072 China; ^2^ Shi‐changxu Innovation Center for Advanced Materials Institute of Metal Research Chinese Academy of Sciences Shenyang Liaoning 110016 China; ^3^ Sichuan Province All‐electric Navigation Aircraft Key Technology Engineering Research Centre Guanghan 618307 China; ^4^ Henan Key Laboratory of High Performance Carbon Fiber Reinforced Composites Institute of Carbon Matrix Composites Henan Academy of Sciences Zhengzhou 450046 China

**Keywords:** fibrous construction framework, ice adhesion, icephobic surface, interfacial elastic instability, undulatory interfacial stress

## Abstract

Icephobic surfaces offer a passive protection strategy against icing hazards in industrial applications. However, the improvement of surface icephobicity is normally accompanied by a compromise in mechanical properties and restrains the practical application of current icephobic surfaces/materials. Here, a generic strategy for icephobic material design is reported to address this conflict through interfacial elastic instability prompted by the synergistic integration of fibrous construction (FC) frameworks with icephobic polymers. The FC‐based material structures could induce periodically localised/amplified stress at the ice/solid interface, leading to interfacial elastic instability and facilitating effective ice detachment. Low ice adhesion strength is achieved, while the tensile strength of the materials is enhanced by 19 times more than the polymer matrix due to the reinforcement from the FC frameworks. Based on the serrated features of the shear forces and numerically simulated interfacial stress distribution, a new theoretical model is established to interpret the nature of interfacial elastic instability and undulatory interfacial stress. This work provides fundamental insights for designing high‐performance icephobic materials that transcend traditional performance trade‐offs, advancing both surface science and materials for practical ice mitigation.

## Graphical abstract

To address the inherent trade‐off between icephobicity and the mechanical strength of icephobic materials, a new icephobic material design strategy is proposed by integrating fibrous construction frameworks and icephobic polymer. The structure prompts interfacial elastic instability and the subsequent undulatory interfacial stress at ice/solid interfaces maintains low ice adhesion strength (6.8 kPa) and increases the tensile strength up to 19 times that of the icephobic polymer matrix.

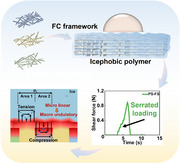

## 1 Introduction

Industrial and commercial activities often suffer from serious safety and economic challenges due to surface icing hazards in cold regions.^[^ ^1^ ^]^ Passive ice protection using icephobic surfaces is a potential solution to solve the icing problem with the advancement of zero energy consumption, surface adaptation, and low structural burden.^[^ ^2^ ^]^ The intrinsic principle of icephobic surface is delaying surface ice nucleation and/or weakening the ice/solid adhesion by optimising surface microstructures,^[^ ^3^ ^]^ deploying low‐modulus elastomers,^[^ ^4^ ^]^ and/or even introducing a slippery liquid layer^[^ ^5^ ^]^ at the ice/solid interface.

The popular topics about icephobic surfaces, such as elastomer‐based coatings (multi‐phase polymer,^[^ ^6^ ^]^ slippery oil‐infused polymer,^[^ ^7^ ^]^molecular‐brush),^[^ ^8^ ^]^ bio‐inspired superhydrophobic icephobic surfaces,^[^ ^9^ ^]^ and photothermal coatings,^[^ ^10^ ^]^ mainly focus on the achievement of excellent icephobic performance with extended icing time and/or low ice adhesion strength.^[^ ^11^ ^]^ However, the enhancement of the icephobicity is often accompanied by a compromise in the mechanical properties of the coating materials, which leads to the poor stability of ice repellency in harsh cold environments.^[^ ^12^ ^]^ For example, the stress‐induced ice removal mechanisms are mainly based on the mismatch of moduli between the ice and the materials, and a lower material modulus would be favourable to reduce ice adhesion strength.^[^ ^13^ ^]^ The increase in coating thickness can further reduce the ice adhesion due to the enhancement of coating elasticity.^[^ ^14^ ^]^ However, the low modulus easily causes the deterioration of mechanical properties, leading to poor resistance to external high‐velocity droplets/particles.^[^ ^15^ ^]^ Researchers have attempted to improve the mechanical strength of icephobic coatings by modifying the polymers with hard molecular chains,^[^ ^16^ ^]^ introducing high‐strength fibres/particles into polymer resins,^[^ ^17^ ^]^ etc. However, a fundamental trade‐off exists between mechanical robustness and icephobic performance.^[^ ^18^ ^]^

More recently, efforts to tailor stress distribution at ice/solid interfaces were attempted by developing multi‐phase icephobic structures, such as porous metal‐supported icephobic polymers,^[^ ^19^ ^]^ sub‐structures designed elastomeric polymers,^[^ ^20^ ^]^ low‐interfacial toughness coatings,^[^ ^21^ ^]^ etc. Compared with traditional icephobic coatings/structures like elastic polymer^[^ ^22^ ^]^ or slippery liquid‐infused porous structures,^[^ ^23^ ^]^ these advanced surfaces achieved ultra‐low ice adhesion strength with efficient interfacial crack propagation due to the modulus variance among different phases.^[^ ^24^ ^]^ However, there was a lack of practical methodologies and theoretical guidance to enhance the mechanical properties while maintaining the ice adhesion strength at a low value.^[^ ^25^ ^]^ The trade‐off between icephobicity and mechanical robustness remains a major challenge in the development of deployable icephobic materials, and a universal principle of material design to simultaneously enhance mechanical and de‐icing performance is desperately required.

Reinforcement using fibres is a common approach in polymer composites, but this strategy has not been well adapted to icephobic materials due to different design criteria and targets. The major question still attributes to the conflict of increasing material strength while maintaining icephobicity. Based on the adhesion theory, due to the low modulus of elastic coatings, the external shear force on ice blocks leads to elastic deformation with varied stress distribution (from tensile to compressed displacement) along the ice/solid interface.^[^ ^26^ ^]^ This mechanism restrains the further improvement of mechanical strength. On the other hand, according to the adhesion theory of rigid‐glass prism and confined elastomeric coating, the tangential force leads to the unstable adhesive interface, further resulting in fracturing bubbles at the interface during the gradual detachment. However, this unstable interfacial fracture merely relies on the elasticity of the coating, which can not guide the further development of robust and efficient icephobic structures. Thus, besides the normal low‐modulus concept, the above struggles may be addressed by additional ice removal mechanisms, which have the potential to prompt the interfacial unstable state and enhance the mechanical strength at the same time.

For material reinforcement, 3D fibrous constructions (FC) through the bridging of various fibres would endow materials with high mechanical strength. By adjusting the fibre species,^[^ ^27^ ^]^ distribution and preparation methods,^[^ ^28^ ^]^ the properties and pore structures of FC can be flexibly regulated. Thus, integrating the FC framework with icephobic polymers will be a promising strategy for optimising the mechanical strength and prompting the interfacial unstable state of icephobic materials simultaneously. Compared to simply utilising the elasticity of the pure elastomeric coatings, the local modulus variance created by the FC framework has the potential to prompt unstable elasticity at ice/solid interfaces. This situation will be beneficial to maintain the low ice adhesion strength with the mechanical enhancement of the FC framework.

Herein, a new material design strategy based on the integration of FC framework and icephobic polymer was proposed to address the trade‐off balance between surface icephobicity and mechanical properties of the icephobic materials. It was demonstrated that the local modulus variance created by the FC framework would prompt elastic instability at ice/solid interfaces, which was attributed to an additional ice removal mechanism. The structure promoted undulatory interfacial stress and kept low ice adhesion strength at a low level, while the tensile strength of the materials was significantly increased due to the reinforcement from the FC frameworks. These findings establish a materials design paradigm that successfully reconciles the traditionally competing requirements of mechanical robustness and icephobic efficiency.

## 2 Results and Discussion

### 2.1 Interfacial Elastic Instability and Material Design

**Figure 1:**
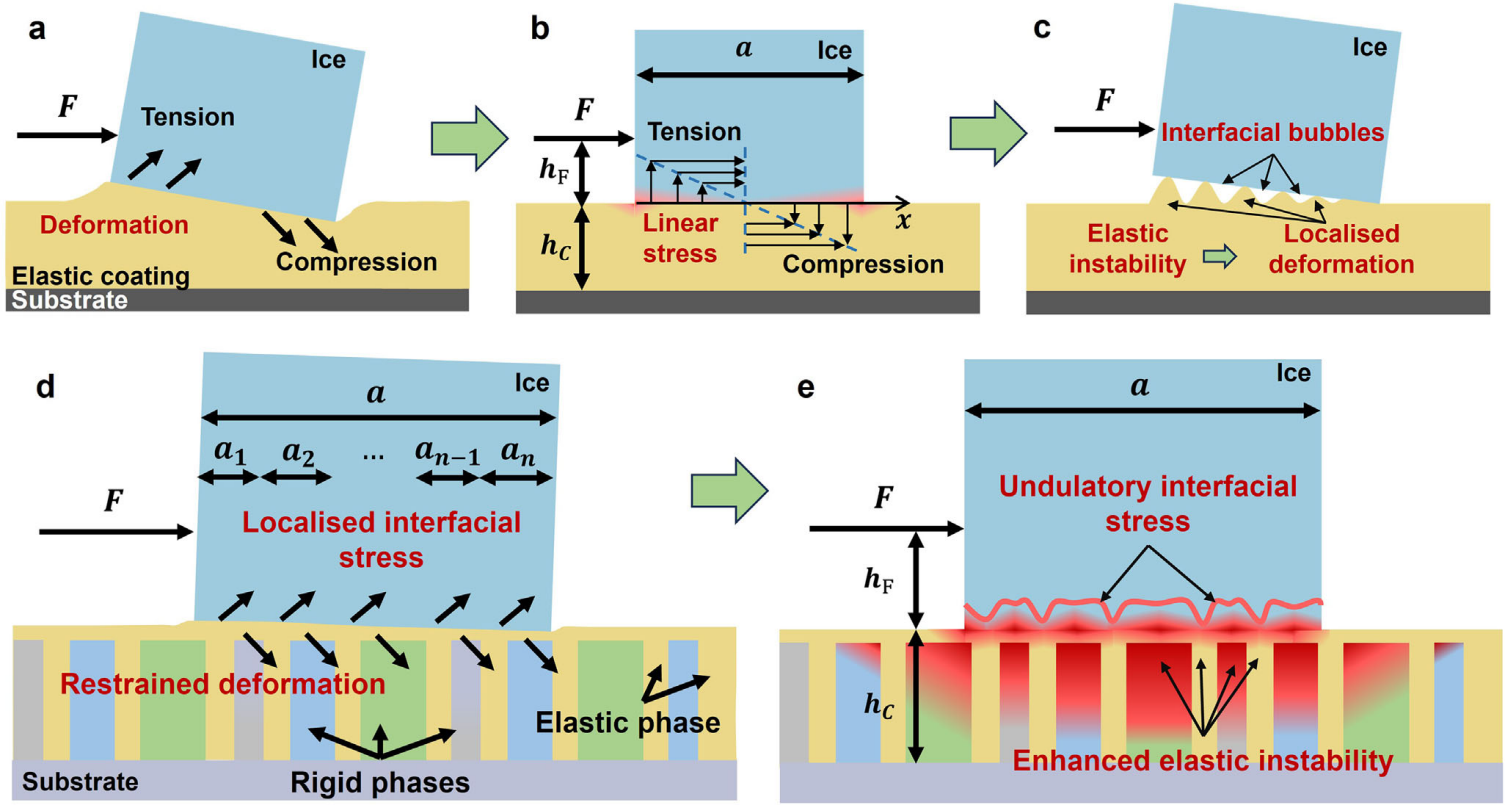
Theoretical adhesive/fracturing model of elastic surface and ice system, reorganised from reference:^[^ ^30^ ^]^ a) elastic deformation under the application of external shear force, b) schematic linear stress distribution at ice/solid interface, and c) hypothetical fracture process with localised deformation and interfacial bubbles, induced by elastic instability at the interface; Proposed theoretical adhesive/fracturing model of multiple‐phase icephobic surface and ice system: d) restrained deformation and localised interfacial stress, caused by the hybrid effect of FC framework and elastomeric phase, e) enhanced interfacial elastic instability and undulatory interfacial stress.

Based on the adhesion theory,^[^ ^26^, ^29^ ^]^ with the application of an external shear force, the adhesion, surface deformation, interfacial stress distribution, and possible fracture behaviour of rigid ice block on an elastic surface are schematically illustrated in **Figure** **1**a–c. Due to the elasticity of polymer coating, the external shear force causes obvious interfacial deformation, in which vertical (to coating surface) displacement happens at the front (tension) and rear (compression) region of the ice/solid contact interface, as shown in Figure 1a. In Figure 1b, the shear force (*F*) is vertically applied on the side surface of the ice block, with a distance of *h_F_* from the coating surface. The coating thickness is defined as *h_c_*, while the length of the ice/solid contact interface is defined as *a*. Chaudhury et al. experimentally observed that part of the adhesive region detached in advance and left bubbles at the interface during the fracture process.^[^ ^30^ ^]^ They explained the model with unstable interfacial elasticity by classifying the coating into a series of ice‐attached springs (as shown in Figure 1c). The detachment was then seen as the orderly break of ice/spring interfaces with linearly varied interfacial stress ($\sigma_{n}∼\frac{\mu \delta }{h_{C}}$, where μ is the shear modulus of the coating) across the interfaces (from the maximum at *x* = 0 to the rare region). Herein, the adhesion stress of the interface is defined as:^[^ ^26^, ^30^ ^]^

(1) $$\sigma_{s}∼\left(\frac{a}{h_{F}}\right)\sqrt{\frac{W_{a}\mu }{h_{c}}}$$

where *W_a_* is determined by the surface energy of materials and μ relates to the elasticity of materials. Further interpretation regarding Figure 1a–c can be found in Section S1 (Supporting Information). Based on Equation (1), the icephobic surface (low ice adhesion strength) is developed by involving low surface energy materials (fluorinated groups) and/or low modulus elastomer (PDMS). Thus, the improvement of icephobicity and mechanical strength of the icephobic surface is conflicting as the lowering material modulus always leads to the weakening of the mechanical resistance. The unstable interfacial elasticity is not controllable and efficient enough that the only determining factor is the elasticity of coatings.

Here, we proposed a strategy to promote an additional ice removal mechanism and induce controllable/efficient interfacial elastic instability and undulatory interfacial stress by integrating a multiple‐phase FC framework and elastomeric phase, schematically illustrated in Figure 1d,e. Due to the supporting effect of the FC framework, the surface deformation was restrained efficiently, as shown in Figure 1d. Then, the ice/solid interface was divided into numerous sections (*a_i_*, *i* = 1 to n) based on the structures of the FC framework. At each section *a_i_*, the local modulus variance between the elastic and rigid phases had the potential to prompt interfacial elastic instability at the ice/solid interface, which led to the periodic localised/amplified stress concentration. The microscopic elastic instability and periodic stress concentration finally led to the macroscopical undulatory interfacial stress, as shown in Figure 1e. The undulatory effect followed the arrangement, size, and modulus of rigid phases in the FC framework. Thus, besides simply relying on the elasticity mismatch of the materials, this additional ice removal mechanism could prompt interfacial elastic instability by optimising the structure of the FC framework. The microscopic elastic instability and macroscopical undulatory interfacial stress offered multiple potential breakpoints that initiated the interfacial cracks much more easily than pure elastomeric coatings. Thus, the integration of the elastomeric phase and FC framework was prone to obtain the lifted balance of icephobicity (interfacial elastic instability) and mechanical properties (fibrous construction reinforcement).

### 2.2 Preparation and Optimisation of the FC Framework

**Figure 2:**
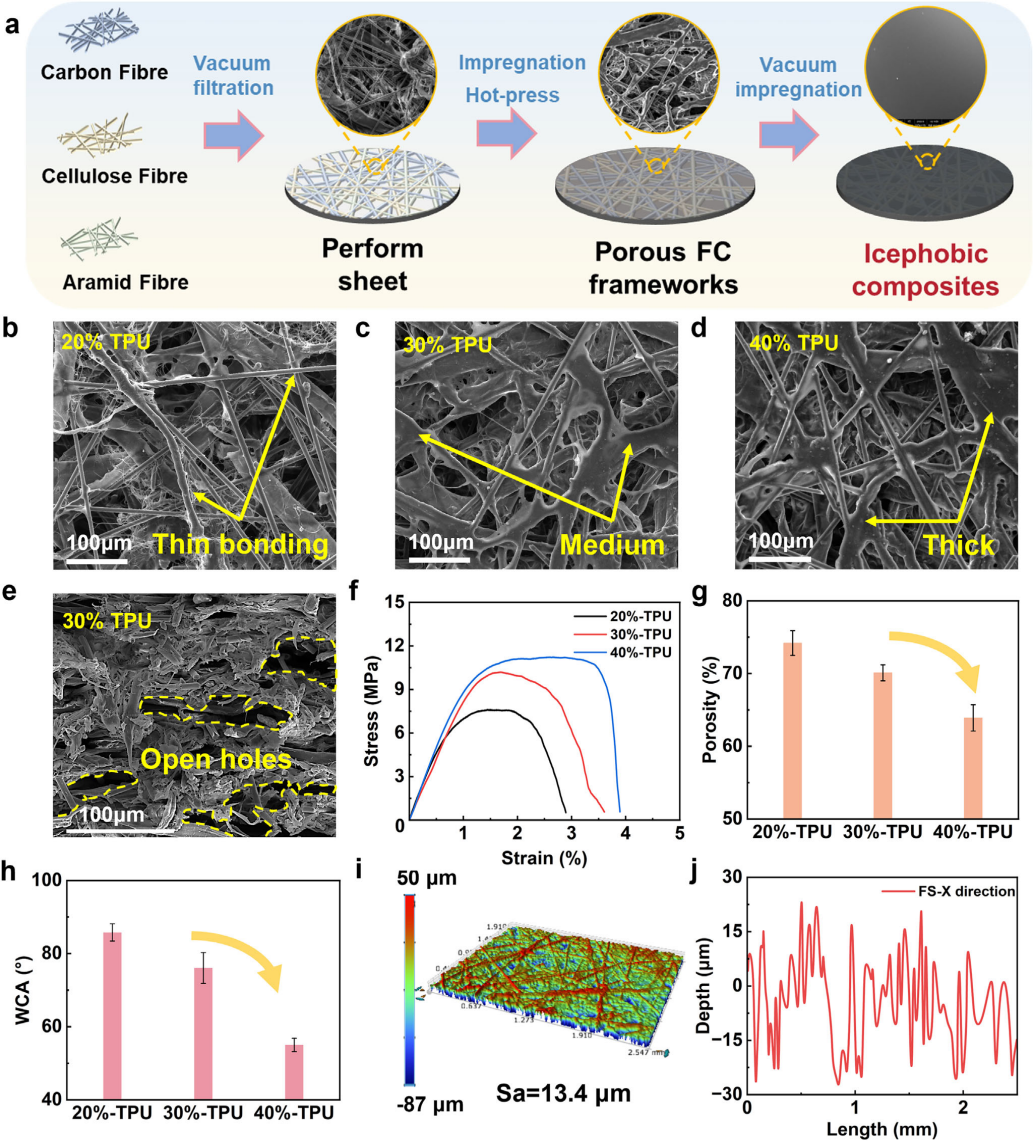
a) The schematic process of preparing icephobic surfaces based on the interfacial elastic instability; surface SEM morphology of porous FC frameworks: b) 20%‐TPU, c) 30%‐TPU, d) 40%‐TPU; e) cross‐sectional morphology of porous FC frameworks with 30% TPU content; f) stress‐strain curves, g) porosity and h) WCA of different FC frameworks; i) 3D contour morphology and j) depth variation in the X direction of 30%‐TPU frameworks.

Based on the above analysis, three kinds of fibres and commonly used icephobic polymers were used to prepare a series of FC frameworks, icephobic surfaces, and control groups (elastic icephobic coatings), followed by the procedure in **Figure** **2**a. In general, two kinds of icephobic surfaces (P‐FS, PS‐FS) and four kinds of control group samples (FS, PS, P‐Al, PS‐Al) were prepared and studied in this paper. Detailed explanations about these abbreviations can be found in **Table** **1**.

**Table 1:** Description and abbreviations of the studied samples.

Classification	Abbreviation	Description
Controlled group	PDMS	Polydimethylsiloxane (PDMS) bulk
	PS	Polydimethylsiloxane (PDMS) bulk swelled by silicone oil
	Al	Al plate
	FS	Fibrous construction frameworks
	P‐Al	Al plate coated with PDMS coating
	PS‐Al	Al plate coated with PS coating
Icephobic surfaces	P‐FS	FS impregnated with PDMS
	PS‐FS	FS impregnated with PS

Figure 2b–d shows the surface morphologies of porous FC frameworks with polyurethane (TPU) contents from 20% to 40%. In general, overlaps and bridging among fibres resulted in the formation of well‐developed pore structures inside. In the low content of TPU resin in Figure 2b, incomplete impregnation resulted in thin bonding between fibres and resin, making the frameworks loose. With the increase of resin content in Figure 2c,d, enhanced and thicker resin bonding happened at the junctions of fibres, reducing the voids and pores in the composite material, thereby making the structure denser and strengthening the inter‐component bonds. The cross‐sectional morphology of FC frameworks (chosen 30%‐TPU as an example) in Figure 2e proved that the resin bonding also occurred inside the structure, with both good connections and plenty of open holes. Mercury intrusion porosimetry results indicated that the 30%‐TPU porous FC framework has a porosity of 73% and an average pore size of 23.42 µm (Section S2, Supportingty Information).

Figure 2f,g depict the alterations in mechanical properties and porosity of porous FC frameworks. With an increase in TPU content in Figure 2f, the maximum load‐bearing capacity of the materials gradually augmented, while the diminished porosity indicated an increase in densification. Due to the similar slope of these curves, there was no notable difference in the elastic modulus with varying TPU resin content. The changes in TPU content primarily enhanced the bonding between resin and fibres to improve resistance to external forces, while fibre structure played an important role in the elastic modulus. Due to the polar groups (urethane and ether bond) in both hard and soft segments of TPU molecular chains, the usage of TPU facilitated hydrogen bonding with water molecules. Thus, the WCA decreased from 85° to 55° in Figure 2h, indicating the degraded hydrophobicity with increasing TPU content. Considering the comprehensive properties, the porous FC framework with 30% TPU content had the best potential and was chosen as the candidate for the following study (labelled as FS). Figure 2i,j illustrate the 3D topography and depth variations along the X direction of FS, respectively. Given the porous and loose surface morphology, the extensive porosity and protruding fibres resulted in a high surface roughness of 13.4 µm, with a pronounced variation in surface depth.

### 2.3 Preparation and Mechanical Properties of Icephobic Surfaces

**Figure 3:**
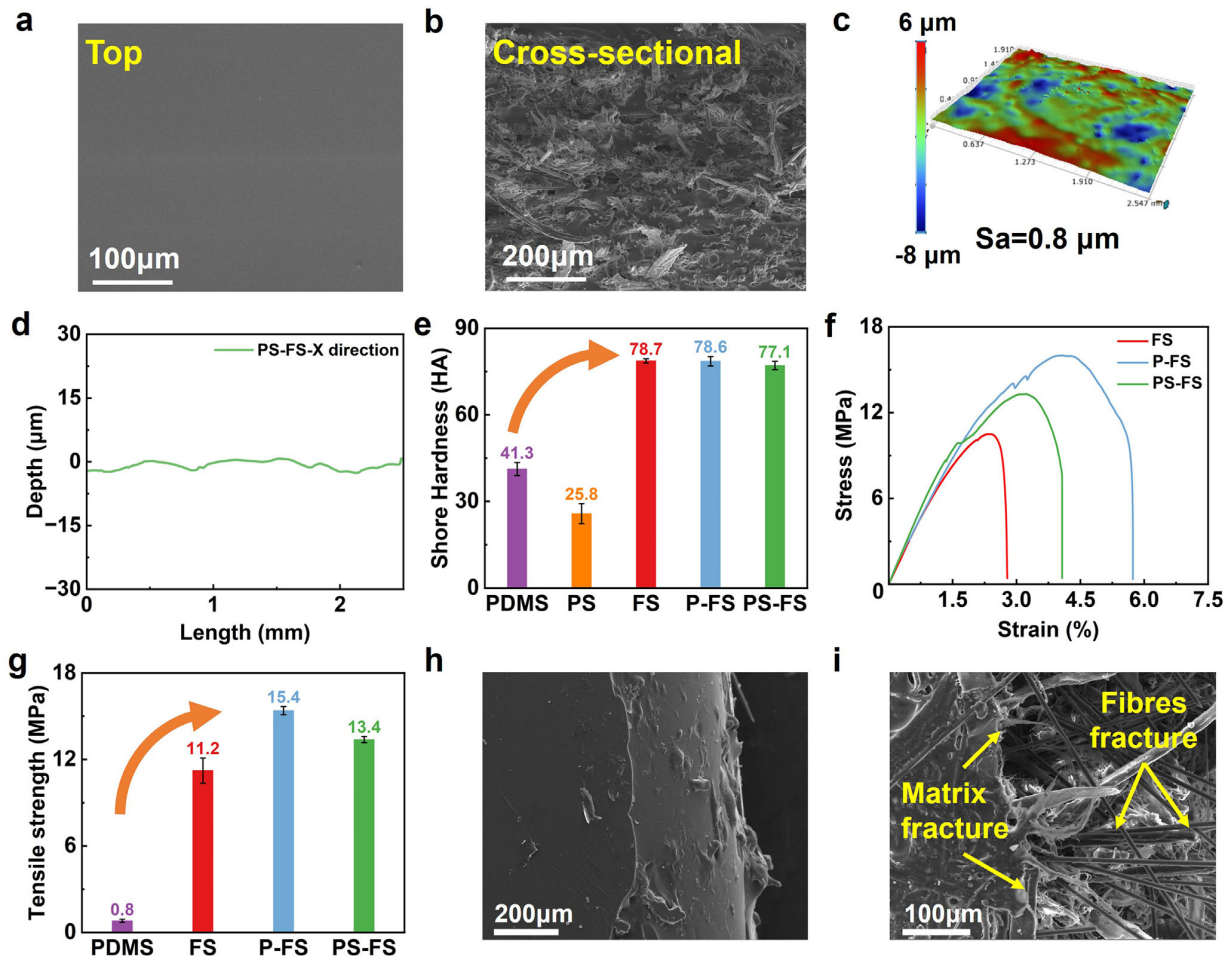
The morphology information of PS‐FS: a) surface morphology, b) sectional morphology, c) 3D contour morphology, and d) depth variation in the X direction; mechanical properties of icephobic materials: e) Shore hardness, f) stress‐strain curves, g) tensile strength; Cross‐section topography after tensile strength tests: h) PDMS; i) PS‐FS.

The icephobic surfaces were finally prepared by vacuum impregnation/curing of icephobic polymers (PDSM and PS) in the frameworks. **Figure** **3**a displays the surface of PS‐FS after infiltration with swelled PDMS with a smooth and even morphology. Figure S2 in (Section S3, Supporting Information) illustrates the porosity change from 82.0% to 21.4% among the perform sheet, FS, and icephobic surface (PS‐FS). The cross‐sectional morphology of PS‐FS Figure 3b showed that internal pore defects were almost filled. Figure 3c,d illustrate the 3D topography and depth variations along the X direction of PS‐FS, respectively. Compared to the rough surface of FS, the surface roughness of PS‐FS significantly decreased to 0.8 µm.

The Shore hardness of PDMS and PS (the mass ratio of PDMS and silicone oil was 2:1) was 41.3±2.2 and 25.8±3.4 HA, respectively, in Figure 3e. The Shore hardness of the polymer with various silicone oil contents can be found in Section S3 (Supporting Information). The Shore hardness of FS was 78.7±0.7 HA, while that of P‐FS was 78.6±1.6 HA, and PS‐FS was 77.1±1.4 HA. The values of icephobic composites were notably higher than those of polymers (PDMS and PS), indicating the mechanical enhancement of the FC framework. The fibrous skeletons supported the whole structure and mitigated the softness of icephobic polymers.^[^ ^31^ ^]^

The tensile strength was 0.8±0.1 MPa for PDMS and 11.2±0.8 MPa for FS in Figure 3f,g. After compositing the icephobic polymers and FC framework, the tensile strength rose to 15.4±0.4 MPa (P‐FS) and 13.4±0.3 MPa (PS‐FS), increased by ≈19 times and 16 times compared to that of the pure PDMS, respectively. The primary reasons for the enhancement were high‐strength fibres and the complete impregnation of PDMS, which filled the internal pores of the material, eliminating significant void defects and improving the interfacial bonding between fibres and resin.^[^ ^32^ ^]^ The fracture surface of PDMS was flat in Figure 3h. The fracture surface of PS‐FS in Figure 3i showed the pulled‐out fibres, which were attributed to the carried stress of the fibrous structure during the tensile process, effectively dispersing and bearing the tensile load. Additionally, PDMS can interact with active groups (such as carboxyl and amide groups) or chemical bonds (such as amide and hydroxyl bonds) on the surfaces of carbon and aramid fibres,^[^ ^33^ ^]^ aiming to promote the interfacial binding of resin and fibres as an effective bridge, further contributing to resisting external damage and improving the tensile strength. However, due to the swelling effect of silicone oil, the oil molecules reside in the interstitial spaces between the PDMS chain segments, leading to the reduction of van der Waals interactions between PDMS molecules, thereby decreasing the force required for intersegmental movement and subsequently lowering the tensile strength.^[^ ^34^ ^]^ Due to the above analysis, the tensile strength of PS‐FS in Figure 3g was slightly lower than P‐FS.

Figure 3f illustrates the stress‐strain curves of FS, P‐FS, and PS‐FS. The increase of curve slopes for P‐FS and PS‐FS proved a higher elastic modulus than FS, while the rise in the maximum load‐bearing capacity demonstrated the enhanced deformative resistance of icephobic composites. More importantly, compared to the smooth tensile curve of FS in Figure 3f and PDMS in SI (Section S3, Supporting Information), P‐FS and PS‐FS showed progressive response characteristics during the tensile process. The hybrid effect of FC frameworks and icephobic polymer leads to complex fracture behaviours during the tensile tests, due to their heterogeneous structures involving FC frameworks, matrixes, and interfaces among various constituents.

The combination of the polymer matrix and FC framework then enhanced the tensile strength. For the de‐icing process with the shear load on the ice, the hybrid effect of FC framework and polymer had the potential to aggravate the elastic mismatch at the ice/solid interface, which promoted the initiation of microcracks, leading to low ice adhesion strength.^[^ ^35^ ^]^

### 2.4 Experimental Verification of Interfacial Elastic Instability during the De‐Icing Process

**Figure 4:**
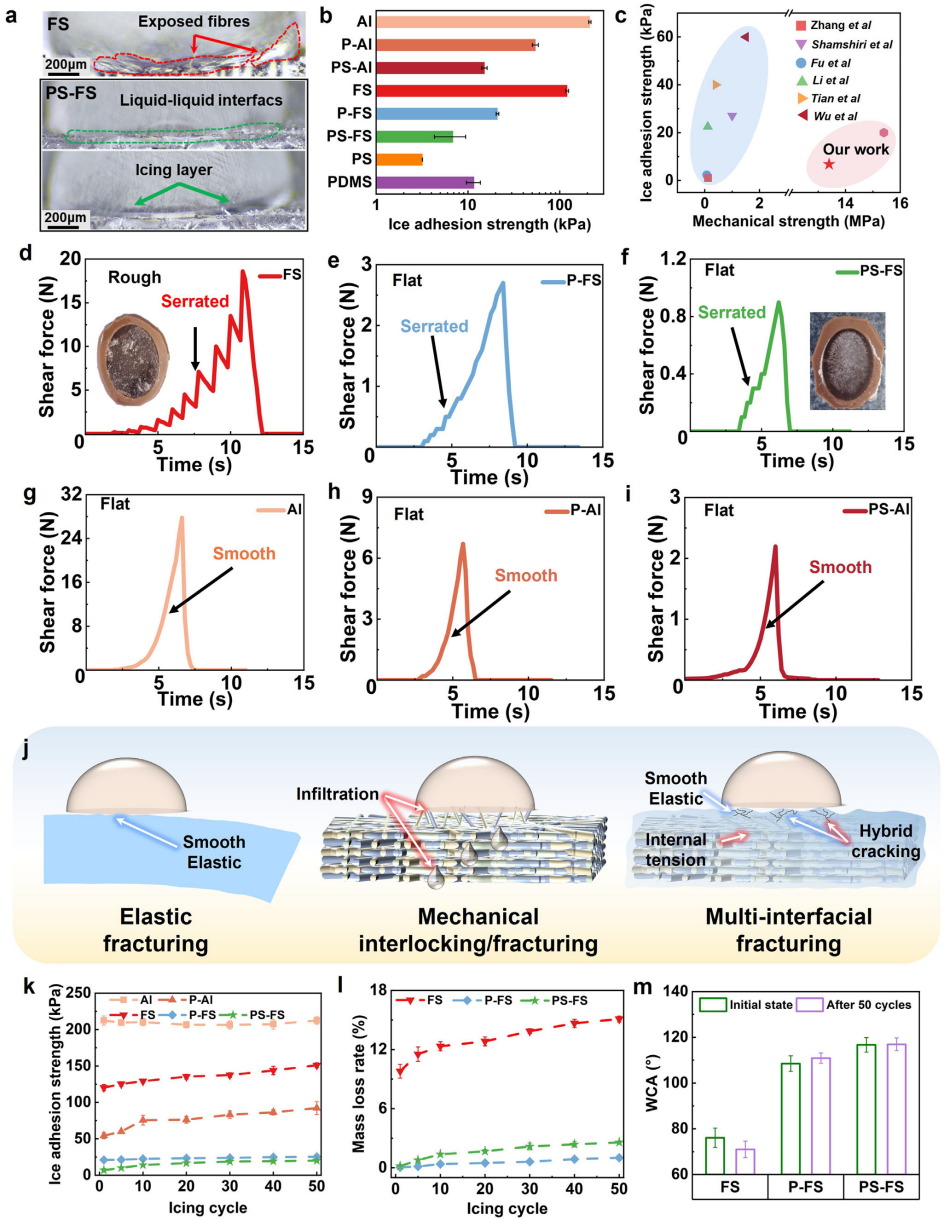
a) Interface state of ice attachment on FS and PS‐FS surfaces; b) comparison of ice adhesion strength of the samples as prepared, tested at −10 °C; c) icephobicity and mechanical robustness performance comparison with other advanced icephobic surfaces; the curves of ice detached shear force of different samples: d) FS, e) P‐FS, f) PS‐FS, g) Al, h) P‐Al, i) PS‐Al; j) schematic illustration of ice detaching processes for elastic polymer, FC framework, and icephobic surfaces with interfacial elastic instability; comparative performance of icephobic surfaces and control group in 50 icing/de‐icing cycles: k) ice adhesion strength, l) mass loss rate; m) WCA of the icephobic surfaces before and after 50 icing/de‐icing cycles.

**Figure** **4**a illustrates the interfacial freezing process on the rough FS surface and smooth PS‐FS surfaces. The surface of FS, characterised by numerous pores and exposed fibres, provided abundant nucleation sites (as depicted in the red dashed line). The formed ice was then mechanically interlocked with rough surface structures. With the addition of a smooth oil layer at the ice/structure interface, droplets exhibited minimal preferential orientation during nucleation, resulting in ice nuclei forming almost simultaneously across the entire interface before spreading upward, as depicted in the green dash. This phenomenon converted the ice‐solid interaction into the ice‐liquid state, eliminating ice pinning points and the mechanical interlocking. Further experimental results and analyses about the wettability, droplet icing behaviour, and the accumulation of frost on the prepared samples can be found in Section S4 (Supporting Information).

Figure 4b shows the comparison of ice adhesion strengths (tested at −10 °C), where the rough FS sample has a high value of 120.2±4.5 kPa. After the integration with icephobic polymers, the ice adhesion strength of icephobic surfaces (P‐FS and PS‐FS) markedly decreased to 20.9±0.7 and 6.8±2.5 kPa, respectively. The values of icephobic polymer bulks were 11.6±2.0 kPa of PDMS and 3.2±0.1 kPa of PS. Figure 4c illustrates the comparison of ice adhesion strength and tensile strength of this study with other references.^[^ ^16^, ^25^, ^36^ ^]^ The proposed materials in this study had comparable ice adhesion strengths with other reported data, while the tensile strength of this work showed a great advantage. The icephobic surfaces in this study demonstrated a lifted balance of mechanical strength and efficient icephobicity. For comparison, the ice adhesion strength at even lower temperatures was also evaluated and summarised in Figure S8 (Supporting Information).

A similar trend also occurred for the control group (Al‐based samples) in Figure 4b, where the ice adhesion strength of Al, P‐Al, and PS‐Al decreased from 212.5±6.3 to 54.2±4.0 kPa and 15.1±1.0 kPa. The reasons were the involvement of low modulus and low surface energy polymer (PDMS), which transferred the ice attachment from the rough metal or fibre surface to the elastic polymer surface. For the oil‐swelled PDMS (PS‐FS and PS‐Al), besides the effect of icephobic PDMS, the existence of an oil layer lubricated the ice/structure interfaces, further lowering the ice adhesion strength. However, the FS‐based samples always showed a lower ice adhesion than that of the Al‐based samples with the same icephobic polymers.

The shear force curves of the ice‐detaching process are summarised in Figure 4d–i. Compared to the smooth force curves of Al‐based samples in Figure 4g–i, the curves of FS‐based samples in Figure 4d–f showed a distinct serrated feature with lower peak force values. The serrated feature of FS (with the high shear force of 18.7 N in Figure 4d) was much more obvious than that of P‐FS (2.7 N) and PS‐FS (0.9 N). The porous and hydrophilic FS surface led to the penetration of surface water/ice into the materials to form strong mechanical interlocking and high de‐icing shear force. Then, the shear force led to the gradual detachment of the ice/FS interface. The detachment happened among fibre/fibre, ice/fibre, and even ice/ice, which caused the serrated variation of the detaching shear force and the rough fracture surface of the detached ice block (Figure S9, Supporting Information).

Compared to the curve of FS in Figure 4d, the serrated feature of P‐FS and PS‐FS in Figure 4e,f was much weaker. The reason was the involvement of icephobic polymers (PDMS or PS), transferring the ice attachment to ice/polymer or ice/oil interfaces. The different modulus between the fibres and polymers caused the interfacial elastic instability. Although the indirect contact between ice and fibre weakened the serrated feature, the icephobic polymers were still effective mediums to conduct the interfacial elastic instability to the ice/polymer interfaces, as the peak shear forces of FS‐based icephobic surfaces (2.7 N of P‐FS and 0.9 N of PS‐FS) were lower than that of Al‐based icephobic coatings (6.7 N of P‐Al and 2.2 N of PS‐Al).

The above discussion proved the effectiveness of the interfacial elastic instability in the reduction of ice adhesion strength. Figure 4j illustrates the schematic interfacial de‐icing process of elastic coating, FC framework, and icephobic surfaces. The detachment of ice from elastic coatings followed the general fracture principle in Figure 1a,b. With the smooth increase of shear force (as shown in Figure 4h,i), the ice detached from the surface when the interfacial stress reached the critical value. The fracture surface of the ice was quite smooth (Figure S9, Supporting Information). For the FC framework, due to the rough and hydrophilic surface properties, the infiltrated supercooled water formed strong mechanical interlocking after freezing. The detachment was accompanied by gradual cohesive failure of both ice and fibres, which led to the serrated shear force curve in Figure 4d, and rough fracture surfaces of ice and FS sample. The roughness of the detached ice bottom and the amount of broken fibres increased with the decrease in temperature (Figure S9a, Supporting Information). Regarding the icephobic surfaces, the interfacial fracture was accomplished by the hybrid effect of the elastomeric polymer and the FC framework. On the one hand, the ice was attached to the elastic polymers, leading to flat fracture surfaces due to the elastic fracture mechanism. On the other hand, the FC framework supported the icephobic polymer, carried internal deformation/tension, and led to the localised and amplified stress concentration at the ice/polymer interface. The above hybrid effect caused the interfacial elastic instability and accelerated cracking with serrated shear force curves in Figure 4e,f, further resulting in the lower fracturing shear force and ice adhesion strength of P‐FS and PS‐FS. The icephobic mechanisms of elastomeric coatings and slippery oil‐swelled polymers have been well‐studied in our previous works.^[^ ^19^, ^37^ ^]^ Thus, the related icephobic performance of PDMS and PS in this study was discussed in Section S5 (Supporting Information).

Figure 4k shows the variation of ice adhesion strength during 50 icing/de‐icing cycles. The ice adhesion strength of Al was always in the range of 200–220 kPa, in agreement with common sense that the Al surface kept consistent surface conditions. For FS, the ice adhesion strength increased from 120.2±4.5 to 151.1±3.3 kPa. This deterioration accorded with the analysis of fracture behaviours of FS, leading to an increase in ice adhesion. For the PDMS‐coated Al (P‐Al), the ice adhesion strength increased from 54 to 90 kPa, which proved that the elastic icephobic polymer could not resist the repeated icing/de‐icing cycles. In Figure S10a (Supporting Information), the ice adhesion strength of various PS‐impregnated V‐PS‐FS also obviously degraded from 3.2±0.1 kPa to higher than 20 kPa. However, for the polymer‐impregnated icephobic surfaces (P‐FS, PS‐FS), the ice adhesion strengths merely increased from 20.9±0.7 and 6.8±2.5 kPa to 25.3±1.4 and 18.9±0.9 kPa, respectively. The improved durability proved that the porous FC frameworks protected the impregnated icephobic polymers by carrying the majority of the external and internal stress, further enhancing the long‐term de‐icing stability.

After 50 icing/de‐icing cycles at −10 °C, the mass loss rates were 15.1% for FS, 1.02% for P‐FS, and 2.57% for PS‐FS in Figure 4l, respectively. Compared to the huge loss rate of other PS‐FS in Figure S10b (Supporting Information) and other references,^[^ ^38^ ^]^ the icephobic surfaces in this study had excellent retention of lubricant, further verifying the advances of icephobic surfaces with the effect of interfacial elastic instability in achieving long‐term de‐icing performance and robustness. With the impregnation of PDMS with silicone oil, the ice adhesion strength of the multiple‐phase icephobic material was kept at less than 20 kPa, and the mass loss rate was less than 3%, indicating an impressive de‐icing performance.

In Figure 4m, the WCA of P‐FS and PS‐FS were kept at the same levels after repeated de‐icing tests. Furthermore, there were no significant changes in the surface morphology of PS‐FS after 50 icing/de‐icing cycles, as shown in Figure S10c (Supporting Information). The abrasion and chemical resistance were also evaluated in this study to explore the environmental resistance of the icephobic surfaces, as given in Section S6 (Supporting Information).

### 2.5 Finite Element Analysis of Interfacial Stress Distribution

**Figure 5:**
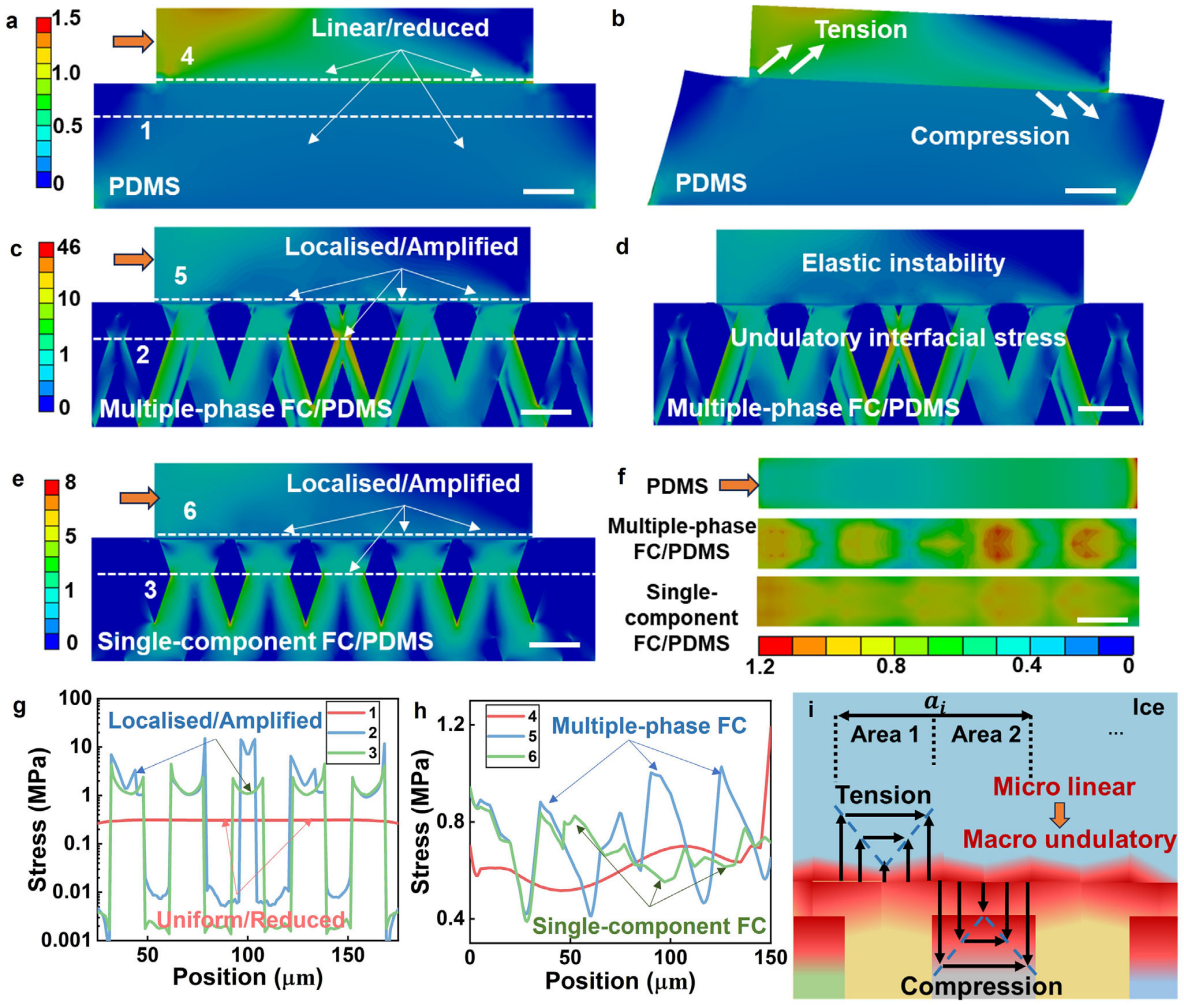
a) Induced stress and b) deformation of the ice and the PDMS coating; c) induced stress and d) deformation of the ice and multiple‐phase FC/PDMS; e) induced stress of ice and single‐component FC/PDMS system (pure cellulose fibre with a radius of 8 µm); f) induced stress on ice bottom surfaces (contacting to icephobic coating and surfaces); g) stress distribution along the select lines inside the materials (lines 1, 2, and 3 in Figure 5a,c,e); h) stress distribution along the select lines of ice bottom surfaces (lines 4, 5, and 6 in Figure 5a,c,e); i) schematic icephobic surface/ice model of the section *a_i_*. All the scale bars represented the length of 20 µm and the external shear loads were 1 MPa.

To seek out the fundamentals of interfacial stress instability and undulatory interfacial stress, six icephobic surfaces/ice integrating models were established to analyse the interfacial stress concentration, as shown and interpreted in Section S8 (Supporting Information). With a shear load of 1 MPa on the ice side surface, the stress distribution inside the icephobic materials and ice bottom surfaces was analysed by FEA and summarised in **Figure** **5**. Figure 5a,c,e illustrate the general stress distribution from the side view among the whole models. Compared to the uniform stress of the polymer/ice model in Figure 5a, the involvement of the FC framework in Figure 5c,e led to the localised and amplified stress concentration at the region of ice near ice/composites interfaces. The periodic stress concentration led to the interfacial elastic instability as undulatory interfacial stress at the ice/composites interfaces, where the undulatory features were along with the structures of fibres. These simulated trends of ice/structure adhesion accorded with the theoretical analysis in Figure 1c,d, in which the involvement of the FC framework optimised the interfacial stress distribution by interfacial elastic instability. Through the comparison between Figure 5b,d, the deformation states were also accorded with the analysis in Figure 1a,c, in which the FC framework restrained the deformation by carrying the majority of the external shear load and internal stress. This phenomenon illustrated that the application of the FC framework was beneficial in enhancing the resistance to external shear loads of composites.

The stress distribution of PDMS coatings in Figure 5a was linear and reduced with values of less than 1 MPa, while much more obvious localised and amplified stress (even larger than 40 MPa) occurred at the multiple‐phase FC frameworks in Figure 5c. The above facts were clearer in Figure 5f of ice bottom surfaces (in contact with icephobic coating and surfaces). When the ice attached to the pure polymer coatings, the general stress was distributed evenly among the ice‐attaching surface with the values (in the range from 0.5 to 0.7 MPa) lower than the 1 MPa shear loading, while the localised stress merely happened on the very end of the edge. For the results for icephobic surfaces with FC frameworks, the stress concentration showed periodically localised and amplified features at the fibre pitching regions with higher values (from 0.7 to 1.2 MPa). This fact proved that the icephobic surfaces were more efficient in inducing interfacial stress concentration, which had the potential to initiate and propagate cracks at ice/composites interfaces with lowered ice adhesion strength.

From the comparison between Figure 5c,e, the multiple‐phase FC framework had desirable stress localisation with higher stress values among the whole framework than those of the single‐component FC framework (pure cellulose fibre with a radius of 8 µm). The better stress concentration amplified the stress variance between the FC framework and elastomeric polymer, which was prone to prompt the stress difference at the ice/structure interface with better icephobic performance. Figure 5g,h presented the stress distribution along the selected lines inside the materials (marked by white dash lines 1, 2, and 3 in Figure 5a,c,e) and ice/structure interfaces (marked by white dash lines 4, 5, and 6 in Figure 5a,c,e). First of all, the line stress distribution of the FC framework‐supported icephobic surfaces showed much more clearly localised and amplified effects than that of pure polymer in both icephobic materials and ice/structure interfaces, leading to more effectiveness in lowering ice adhesion strength. In Figure 5g about the stress distribution of ice/composites interfaces, the single‐component FC framework (pure cellulose fibre with a radius of 8 µm) showed much lower stress at the pitching region, while the multiple‐phase framework amplified the stress values of the pitching regions. Compared to the single‐component framework (pure cellulose fibre with a radius of 8 µm) supported icephobic surface, the multiple‐phase fibre surface showed better periodic stress localisation and amplification at the ice/structure interface in Figure 5h. Thus, the compositing of elastic polymer and multiple‐phase FC framework was proved to acquire the best interfacial elastic instability and structural stability when the external shear load was applied on the surface attached ice block. To further explore the influence of fibre modulus, radius, and the porosity of FC framework on interfacial elastic instability and stress undulation, three additional simulated models were established for finite element analysis. The related results and discussion are given in SI (Figure S12, Supporting Information).

Different from the linear stress distribution of elastic coating, the proposed interfacial elastic instability and undulatory interfacial stress require a specific theoretical explanation. Thus, based on the width of the fibre and adjacent polymer in the icephobic surface, the ice/composite contact interface (length of *a* in Figure 1d) was cut into section *a*_1_ to *a_n_*. A theoretical model of the section *a_i_* was established in Figure 5i to analyse the interfacial stress. According to the results of FEA in Figure 5c–h, the stress localisation and amplification mainly occurred at the pitching regions where the polymer was supported by fibres. The pure polymer pitching regions showed relatively lower stress values. Thus, the section *a_i_* was further divided into Area 1 and Area 2 in Figure 5i. In Area 1, instead of the gradually linear changes from tension stress to compression stress in Figure 1b, the FEA results illustrated that the stress decreased to the minimum value at the middle position, then increased to the end position of Area 1. A similar trend was also observed in Area 2. The difference was the vertical displacement, in which Area 1 only had the tension effect, while the compression effect happened in Area 2. Then, the interface stress of Area 1 and Area 2 can be written as:

(2) $$\sigma_{a_{i1}}∼\left(\frac{a_{i1}}{h_{F}}\right)\sqrt{\frac{W_{a}\mu_{a_{i1}}}{h_{c}}}$$

(3) $$\sigma_{a_{i2}}∼\left(\frac{a_{i2}}{h_{F}}\right)\sqrt{\frac{W_{a}\mu_{a_{i2}}}{h_{c}}}$$

where *a*_*i*1_ and *a*_*i*2_ represented the length of Area 1 and Area 2, $\mu_{a_{i1}}$ and $\mu_{a_{i2}}$ were the shear moduli of Areas 1 and 2. Note that the value of $\sigma_{a_{i1}}$ and $\sigma_{a_{i2}}$ were much less than the interface stress of icephobic coating in Figure 1b, due to the relationship of *a*_*i*1_<<*a* and *a*_*i*2_<<*a*. This trend was accorded with the results of serrated load‐time curves in Figure 4e,f. Part of the section *a_i_* detached from ice with quite low values of shear load when the interfacial stress reached to critical value of $\sigma_{a_{i}}$, leading to a drop in shear load. With the continued de‐icing process, the shear load increased until the next detachment of another section *a_i_*. Then, the load‐time curves showed macroscopically serrated features.

More importantly, due to the hybrid effect of polymer and fibre in Area 2, the modulus $\mu_{a_{i2}}$ was larger than that of Area 1, resulting in the relationship of $\sigma_{a_{i1}}$ < $\sigma_{a_{i2}}$. This fact prompted the elastic instability and adhesion stress difference in the section *a_i_*, which accelerated the crack initiation at the joint between Area 1 and Area 2, further leading to the lowered ice adhesion strength. Thus, although the adhesion stress was linearly distributed at each micro area, the modulus differences of each area caused the stress distinction, leading to the macroscopical interfacial elastic instability with undulatory interfacial stress.

The above analysis proved the nature of the interfacial elastic instability in the reduction of ice adhesion strength, leading to numerous periodic undulatory interfacial stresses. This phenomenon was prone to trigger crack initiation due to the hybrid effect of icephobic polymer and multiple‐phase FC framework. The FC frameworks also carried the majority of induced stress and protected the impregnated icephobic polymers with better mechanical resistance and longer de‐icing lifetime.

## 3 Conclusion

This study presents a new material design strategy to resolve the inherent trade‐off between surface icephobicity and mechanical properties of icephobic materials. By harnessing interfacial elastic instability to promote undulatory stress at ice/solid interfaces, we developed mechanically robust icephobic materials through the synergistic integration of a multiphase FC framework with icephobic polymers. Under the external shear force, the local modulus variance created by the FC framework would prompt unstable interfacial elasticity, which was attributed to an additional ice removal mechanism and was beneficial to maintaining low ice adhesion strength.

The reinforcing effects of the multiphase FC framework and its capability to facilitate the detachment of ice via unstable elasticity have been verified via experimental results and numerical simulation. Low ice adhesion (6.8±2.5 kPa) of the icephobic materials has been achieved, while a 19‐fold enhancement in tensile strength compared to pure polymers is demonstrated, alongside the exceptional environmental stability and de‐icing durability. A new theoretical model was established to elucidate the ice removal mechanisms on the icephobic materials. The correlation between microscale elastic instability and macroscale periodic undulatory interfacial stress was fully discussed, revealing the fundamental principles governing the concurrent optimisation of mechanical strength and efficient icephobicity. This work provides new insights for designing robust icephobic materials while advancing both theoretical understanding of surface adhesion and practical solutions for ice mitigation technologies.

## 4 Experimental Section

### RAW Materials

The carbon fibres (Jilin Jiyan High Technology Fiber Co., Ltd., China), aramid fibres (Dupont, USA), cellulose fibres (Russian Ilim Group, Russian), polyurethane (Estane 2013‐80A, Dongguan Zhongyuan Plastic Materials Co., Ltd., China), polydimethylsiloxane (PDMS Sylgard 184, Dow Corning Corporation, USA), Ethyl acetate, N, N‐Dimethylformamide and silicone oil (Aladdin Industrial Co., Ltd), and aluminium plate 6061 with dimensions of 50 mm × 20 mm × 1 mm (Shenzhen Rui Dewei Co., Ltd., China) were used as received without further purification.

### Sample Preparation

Carbon fibres, aramid fibres, and cellulose fibres were stirred in water until homogeneous dispersion. The evenly dispersed slurry was poured into the paper‐making machine to obtain the wet preform sheet after vacuum filtration and oven drying. The dried preform was immersed in 7 wt.% polyurethane elastomer solution (dissolved in N, N‐dimethylformamide), dried at room temperature, hot pressed, and cured to obtain the porous FC frameworks. The icephobic precursors (mixtures of PDMS elastomer and silicone oil) were obtained by stirring PDMS (10:1 ratio of parts A and B), silicone oil, and ethyl acetate at 2000 rpm for 5 min in a high‐speed defoamer. The porous FC frameworks were then immersed in various icephobic precursors with a vacuum of 0.1 bar for 30 min, heated at 80 °C for 60 min, and finally cured at 110 °C for 360 min to get the icephobic surfaces. For reference, the ultrasonically cleaned Al sheets were also immersed in the various icephobic precursors to prepare metal‐based coating samples (the same thickness as icephobic surfaces). The thickness of all icephobic elastomer coatings was ≈150 µm.

### Structural Characterisation

The micromorphology of the samples was examined by scanning electron microscope (SEM, Czech Tescan VEGA3). The surface profiles and surface roughness were measured through a 3D surface profiler (Keyence VR6000, Japan). Shore hardness was measured using the LXD digital shore hardness tester. Six individual measurements were performed for each sample to ensure accuracy and reduce existing errors. The tensile strength tests were performed on an electronic universal testing machine (CMT5504, China, see details in Section S8, Supporting Information). The pore structure, including pore size distribution, average pore size, and porosity, was characterised using the American AutoPore IV 9500 mercury porosimeter under high pressure based on the Washburn equation ($r=\frac{-2\sigma \cos\theta }{P}$), where *P* is the pressure of high‐pressure intrusion, N m^−2^; *r* is the pore radius, nm; *σ* is the surface tension of Hg, N m^−1^; *θ* is the contact angle of the samples.

### Wettability Evaluations

Static water contact angle (WCA), oil contact angle (OCA, silicone oil), dynamic contact angles (advancing and receding CA, θ_adv_, and θ_rec_), and contact angle hysteresis (CAH) was measured using a contact angle goniometer (CA 200S, China) with a pumping out or absorbing rate of 2 µL s^−1^. All the tests were performed 5 times at different positions to calculate the averages.

### Anti/De‐Icing Tests

Water droplet icing tests were conducted in a cold chamber with a temperature of −20 °C and a relative humidity of 40% without airflow. All the samples were placed in the chamber to cool down to −20 °C in advance. Then, a deionised water droplet (volume of 6 µL) was added to the surface to initiate the icing process. The whole icing process was recorded by a digital video camera to ascertain the duration and phenomena of the icing process. Anti‐frosting tests were conducted in the same cold chamber (composed of a transparent sealed PMMA cover and a cooling plate to form an insulation space) with a controlled aerosol (ultrasonically generated from deionised water) flow at environmental conditions of −10 °C and 98% relative humidity. After 20 min of the pre‐cooling process, the aerosol was input into the chamber for 20 min to evaluate the anti‐frosting performance of the samples. The freezing process of each sample was repeated 15 times, and the frost layer image of the upper surface was captured after each test to investigate the anti‐frosting performance of each sample. The measurement of *ice adhesion strength (*τ) was performed in the cold chamber with an operational temperature of −10 °C (as shown in Section S8, Supporting Information). A semi‐ellipsoidal‐shaped rubber mould with deionised water was used to produce a block of ice on the test surfaces. After a 6 h icing process, uniform ice blocks were formed on each sample for the ice adhesion strength test. The curve of ice detached shear force was recorded by the force gauge (with a moving speed of 2 mm s^−1^) to analyse the fracture process of surface ice blocks.

### Durability Tests

Repeated ice adhesion tests (50 icing/de‐icing cycles) were performed to evaluate the long‐term de‐icing robustness. The mass of the samples before and after the test was also recorded to verify the capacity of oil maintenance. The sandpaper‐wear method was used to estimate the surface abrasion resistance (Section S8, Supporting Information). Place a 100g weight on the surface of 1000 grit sandpaper, with the sample surface in direct contact with the sandpaper surface. Push the sample to move steadily and uniformly in the horizontal direction, with each movement distance of 30mm, and move back and forth 100 times. Record the surface wettability and ice adhesion strength of the worn sample. The chemical resistance was evaluated by placing the samples in HCl (pH = 1) and KOH (pH = 13) solutions for 2 h, after which the surface morphology was analysed and the surface wettability was recorded (Section S8, Supporting Information).

### Numerical Simulation of Interfacial Stress Distribution

To simulate the induced stress distribution at the ice/structure interfaces and inner stress of FC frameworks, six microscopic models were established (as shown in Figure S13a, Section S8, Supporting Information). The upper object was bulk ice, and the bottom part was the PDMS coatings, multi‐phase fibres impregnated with PDMS, and single‐component fibre (pure cellulose fibre with a radius of 8 µm) impregnated with PDMS in Figure S13b–g (Supporting Information). To reflect the stress distribution at ice/solid interfaces, the calculation was performed with the statics module of ANSYS. The ice and coating objects were set as a whole, which means no relative displacement at contacting interfaces under the external loads. A shear loads of 1 MPa were vertically applied to the ice side face. The physical properties of ice, PDMS, and various fibres were obtained from the database of software and references.^[^ ^39^ ^]^ The mesh was divided in a hexahedral manner, and the contact parts were encrypted according to the manner of face size. The total number of grids was 5 0000–50 0000, and the average quality of grids was no less than 0.8 to ensure the calculation accuracy. Considering the real experimental conditions, the constraint was set to a fixed support for the bottom surface of the PDMS coatings. The equivalent stress results were used to reflect the value and distribution of stress of various simulated ice/solid models.

## Conflict of Interest

The authors declare no conflict of interest.

## Supporting information



Supporting Information

## Data Availability

The data that support the findings of this study are available from the corresponding author upon reasonable request.
