# Supplementary material for: Prompting Interfacial Elastic Instability for Effective and Durable Icephobic Surfaces
Source: Adv Sci (Weinh). 2025 Jun 10;12(33):e03855. doi: 10.1002/advs.202503855 (PMC12412557; doi:10.1002/advs.202503855)
Supplement: Supplementary file 1 — Supporting Information [file ADVS-12-e03855-s001.docx]

Supporting information

**Title:** **Prompting Interfacial Elastic Instability for Effective and Durable Icephobic Surfaces**

*Xinyu Fan,* *Jingtong Li, Deyu Yang^*^, Jie Fei^*^, Hejun Li,* *Xianghui Hou^*^*

X. Fan, D. Yang, J. Fei, H. Li, and X. Hou

State Key Laboratory of Solidification Processing, Shaanxi Key Laboratory of Fiber Reinforced Light Composite Materials, Northwestern Polytechnical University, Xi’an 710072, China.

E-mail: [Deyu.Yang@nwpu.edu.cn](mailto:Deyu.Yang@nwpu.edu.cn); [feijiecc@nwpu.edu.cn](mailto:feijiecc@nwpu.edu.cn); [houxianghui@nwpu.edu.cn](mailto:houxianghui@nwpu.edu.cn)

J. Li

Shi-changxu Innovation Center for Advanced Materials, Institute of Metal Research, Chinese Academy of Sciences, Shenyang, Liaoning 110016, China.

D. Yang

Sichuan Province All-electric Navigation Aircraft Key Technology Engineering Research Centre, Guanghan, 618307, China.

X. Hou

Henan Key Laboratory of High Performance Carbon Fiber Reinforced Composites, Institute of Carbon Matrix Composites, Henan Academy of Sciences, Zhengzhou 450046, China.

Keywords: icephobic surface, interfacial elastic instability, undulatory interfacial stress, fibrous construction framework, ice adhesion.

**Section S1: Supplementary of adhesion theory**

Based on the theoretical model in Figure 1a to 1c, the torque balance and the total energy of the system per unit width ($U$) can be written as:[1]

$F\cdot h_{F}=a\int\sigma_{n}xdx$ (S1)

$U=\left( \int_{0}^{a} \int_{0}^{\delta^{*}} \sigma_{n}d\delta dx \right)-W_{a}a$ (S2)

where $\sigma_{n}$ denotes the normal stress at the interface, $x$ represents the distance from the front edge of the ice/coating interface (from 0 to $a$), $\delta$ is the vertical displacement of the elongated coating, $\delta^{*}$ is the maximum displacement at the interface ($x=0$), and $W_{a}$ denotes the work of adhesion. The first term (elastic energy) and the second term (adhesion energy) of Equation (S2) reveal that the interfacial adhesion is determined by the coating elasticity and surface energy.

**Section S2: Supplementary of FC framework structural information**

The control groups in this study are PDMS, PS, Al, FS, P-Al, and PS-Al. The detailed description can be found in Table 1 in the manuscript. PS is a pure polymer elastomer (PDMS swelled by silicone oil). Al has been used as a key structural material in aviation, electric power and other fields. PDMS coating (polymer coating) or PDMS-SO coating (lubricated polymer coating) has been prepared on the Al plate surface. By comparing the above materials with FS-based icephobic surfaces, the proposed anti-icing theoretical concept is further verified. The pore size distribution of the 30% - TPU fibre skeleton was provided in **Figure S1**.


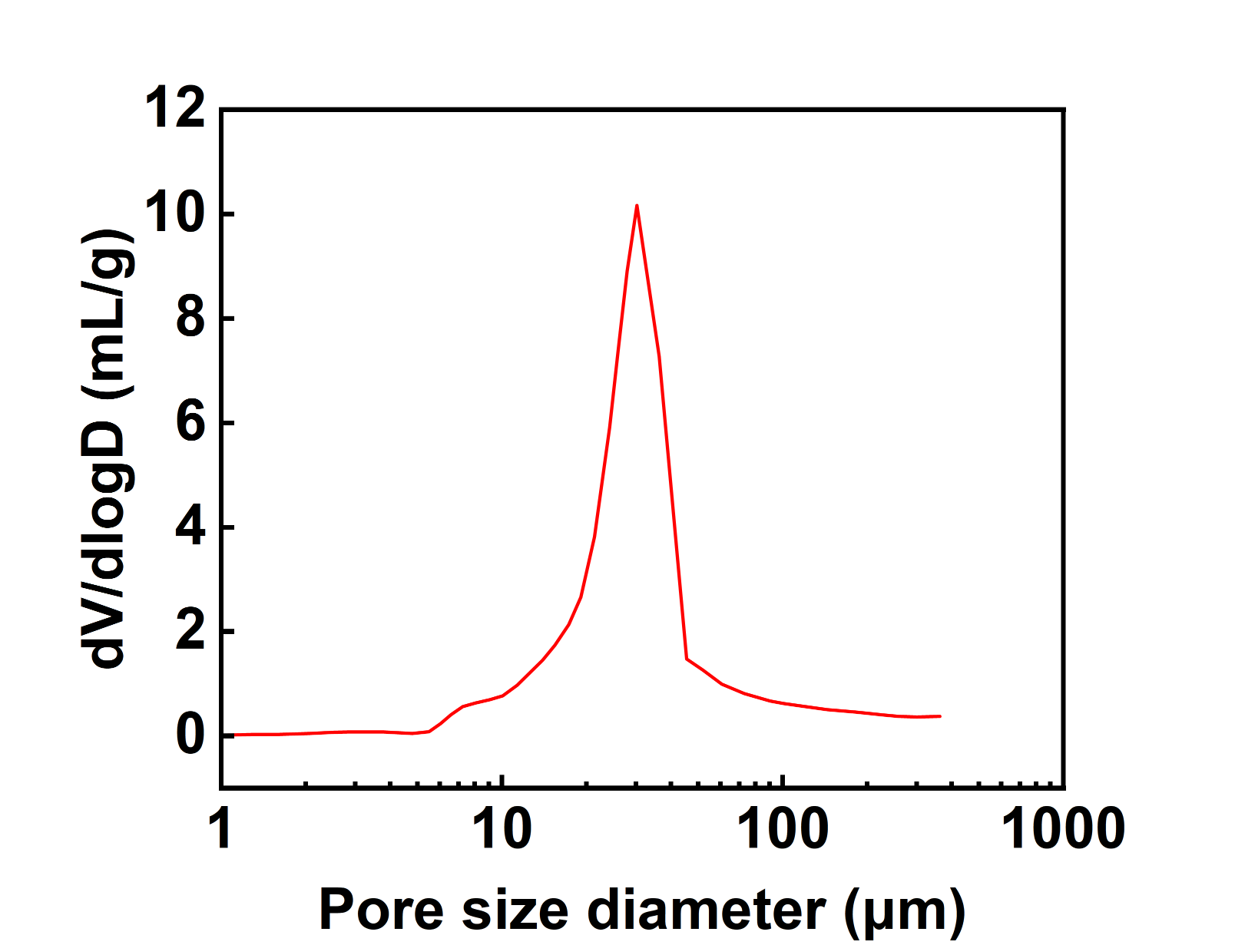


**Figure S1** The pore size distribution of 30% - TPU fibre skeleton

**Section S3: Supplementary of material structural features and properties**


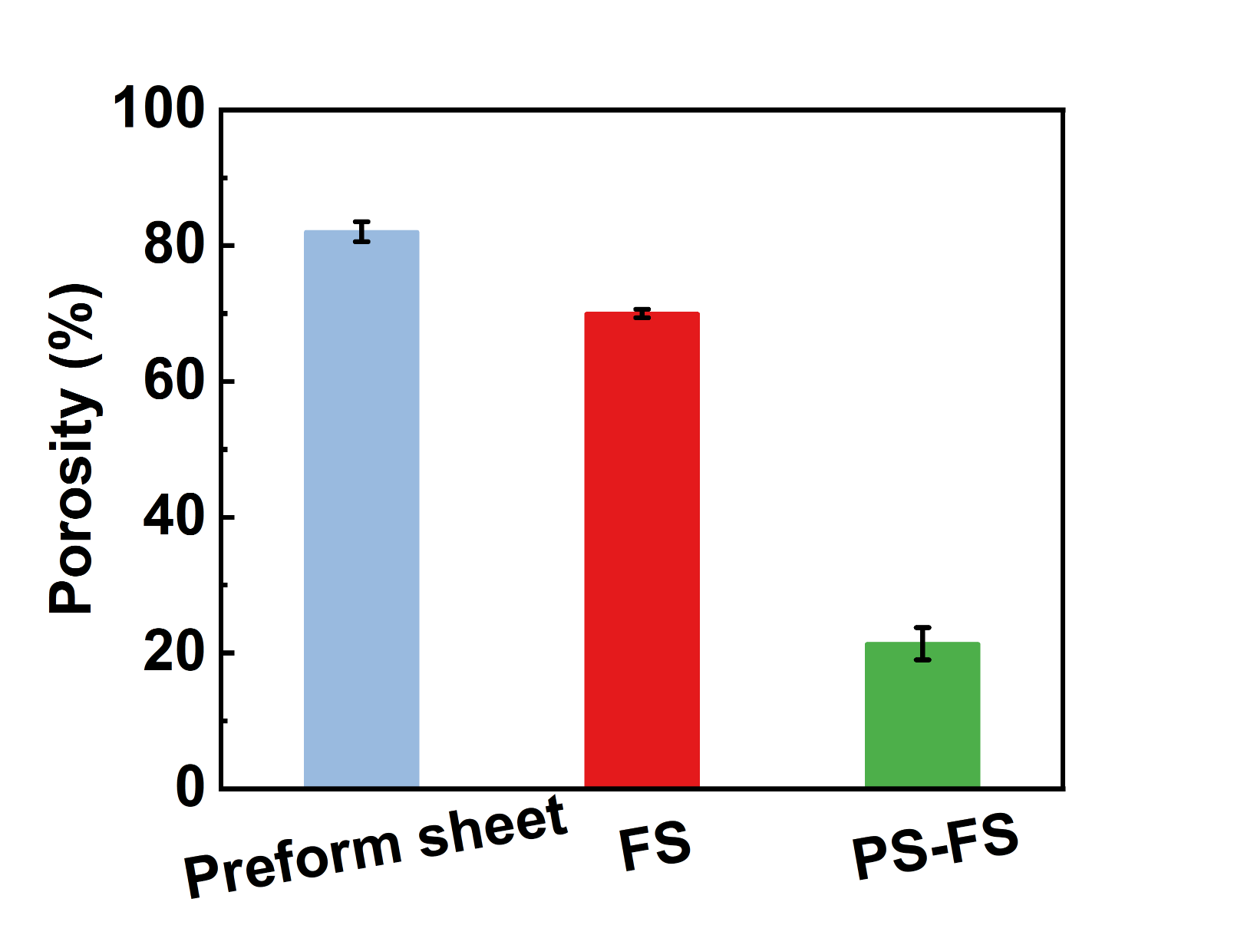


**Figure S2** Porosity evolution in the preparation process of multi-component materials


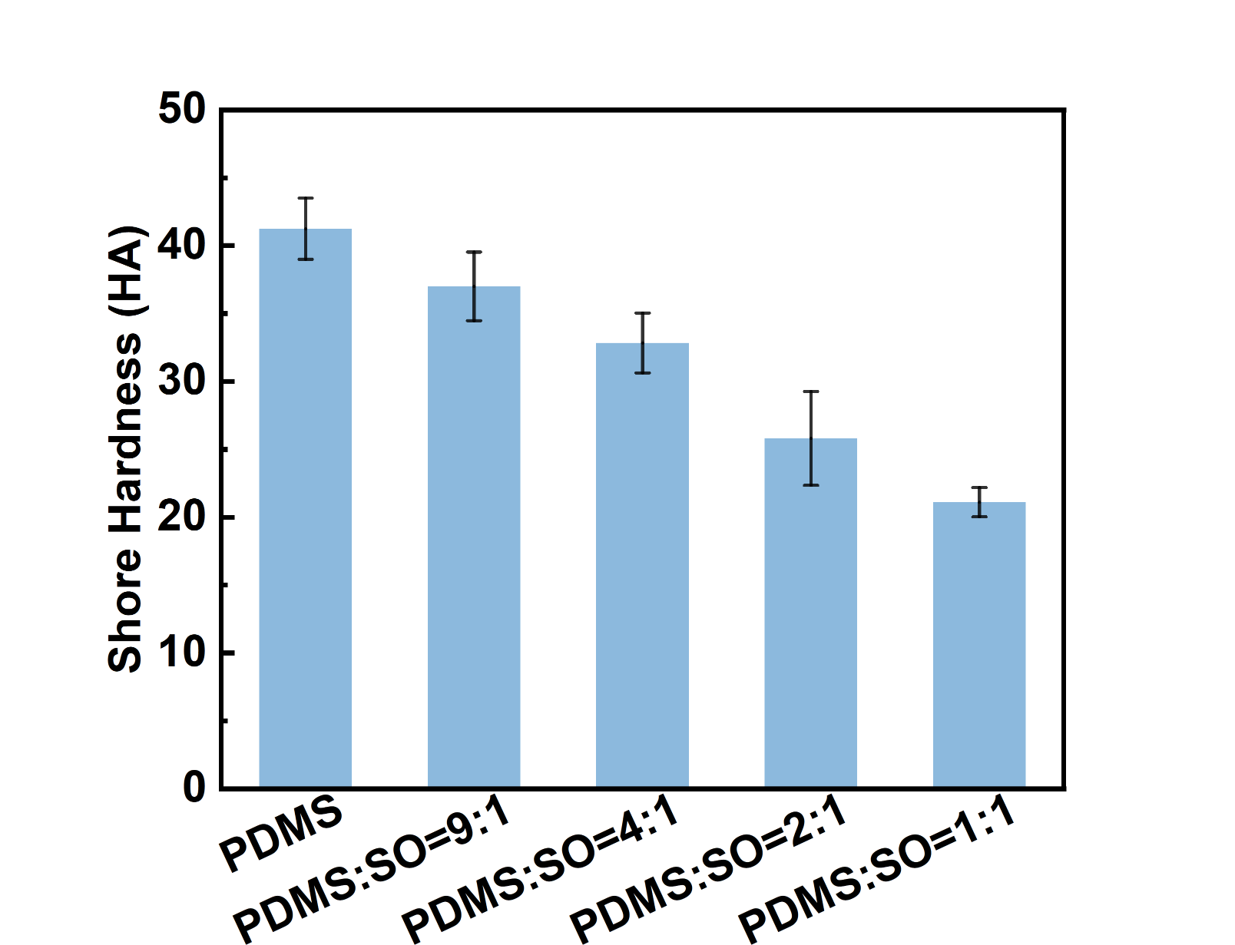


**Figure S3** Shore Hardness of PDMS elastomers infused with different contents of silicone oil

With the addition of internal silicone oil content, the Shore hardness of the polymer gradually decreased, as shown in **Figure S3**. It could be attributed to the swelling effect caused by the addition of silicone oil, which increases the intermolecular spacing within the PDMS macromolecular chains, leading to a decrease in crosslink density and subsequently softening the material.[2]


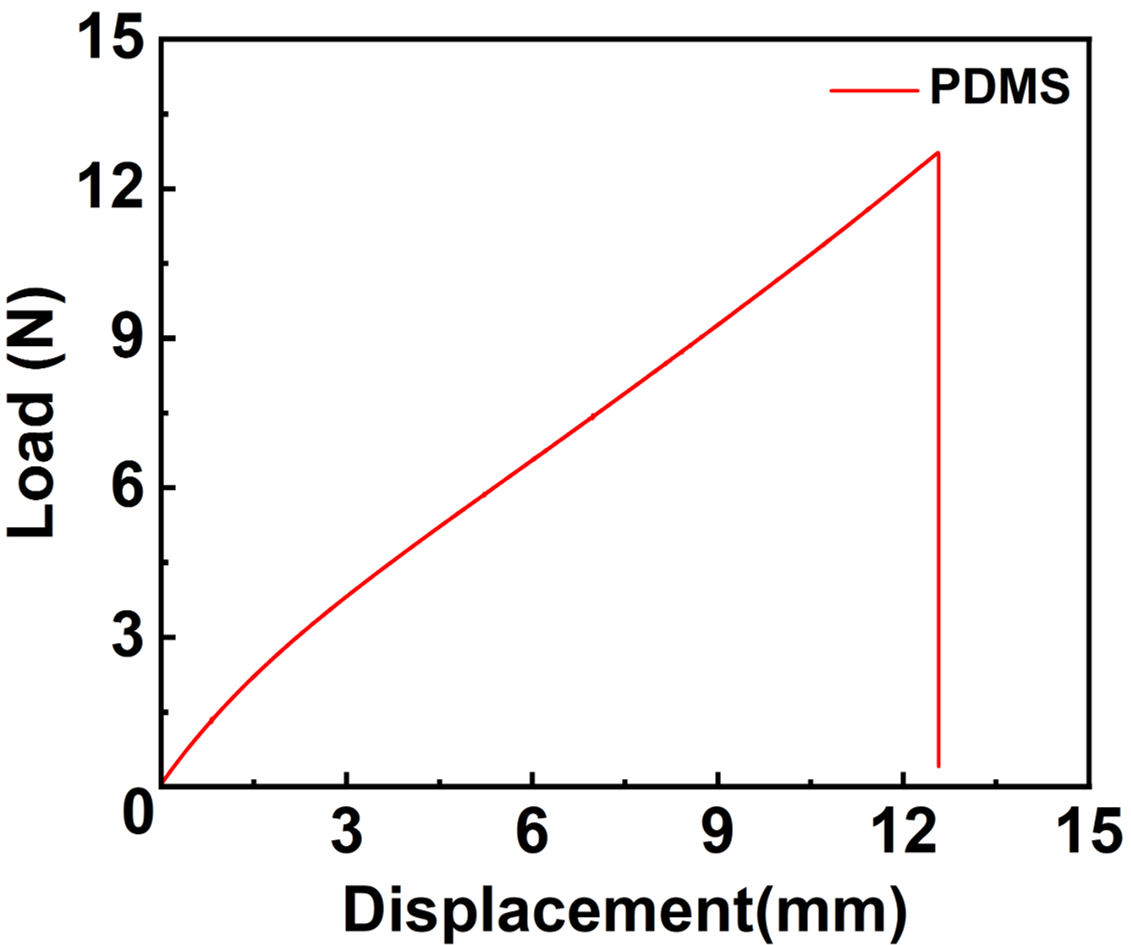


**Figure S4** Tensile load-displacement curve of PDMS with the cross-sectional area of 5.0 mm × 3.3 mm

**Section S4: Analysis of droplet wetting/icing behaviours**

The WCA of FS, P-FS, and PS-FS were 76.0±4.2°, 107.8±2.6°, and 116.7±3.1°, respectively, while the CAH value decreased from 25.1±3.9° to 10.7±0.8°, indicating enhanced hydrophobicity and mobility of water droplets, as shown in **Figure S5a** and **S5b**. The OCA results in **Figure S5c** revealed a decrease from 30° to 22°, signifying improved oil wettability. **Figure S5d** summarised the icing time of supercooled water droplets on various material surfaces. As a reference sample, the water droplet took the shortest time to freeze on the Al plate (12±3 s), primarily due to poor hydrophobicity and rough surface structure. After the assembly of PDMS coating, the icing time for P-Al extended to 50.5±3.5 s, attributed to the improvement of hydrophobicity (transferring the rough metal surface to a smooth polymer surface). When the polymer coating changed to swelled PDMS (PS), the icing time was further extended to 73.3±7.6 s of PS-Al. The presence of a silicone oil layer on the surface reduced the liquid-solid contact area and anchoring points, thereby potentially extending the icing time.[3] A similar trend was also found for the icephobic surfaces with FS frameworks, where the icing time of FS, P-FS, and PS-FS increased from 65.3±8.7 s to 134.2±27.3 s and 315.5±34.6 s, respectively.

The Gibbs free energy for heterogeneous nucleation ($\Delta G_{c}$) and homogeneous nucleation $(\Delta G_{c}^{\text{Homo }})$ were calculated by:[4]

$\Delta G_{c}^{\text{Homo }}=\frac{16\pi\gamma^{3}}{3(\Delta G_{V})^{2}}$ (S3)

$\begin{aligned} \Delta G_{c}=\Delta G_{c}^{\text{Homo }}f(m,x) \end{aligned}$ (S4)

where $\Delta G_{V}$ shows the free energy difference per unit volume, and $\gamma$ represents the interfacial tension between the ice nucleus and liquid. $f(m,x)$ is the geometric parameter of surface wettability and surface topography. $m$ was determined by the surface energy of the interfaces, while $x$ is related to the radius of features of the surface (*R*) and the critical nucleolus radius ($r^{*}$). The presence of the impregnated polymer converted the contact interfaces from rough water/fibres to smooth water/polymers. The smoother interface increased the nucleation free energy barrier with a slower icing process. Compared to the dry icephobic polymer (P-FS), the presence of the oil film on PS-FS transferred the water-solid interface between droplet and PDMS into the water-oil interface, thereby further eliminating potential nucleation sites, inhibiting ice nucleation, and facilitating longer icing time.[3, 5]


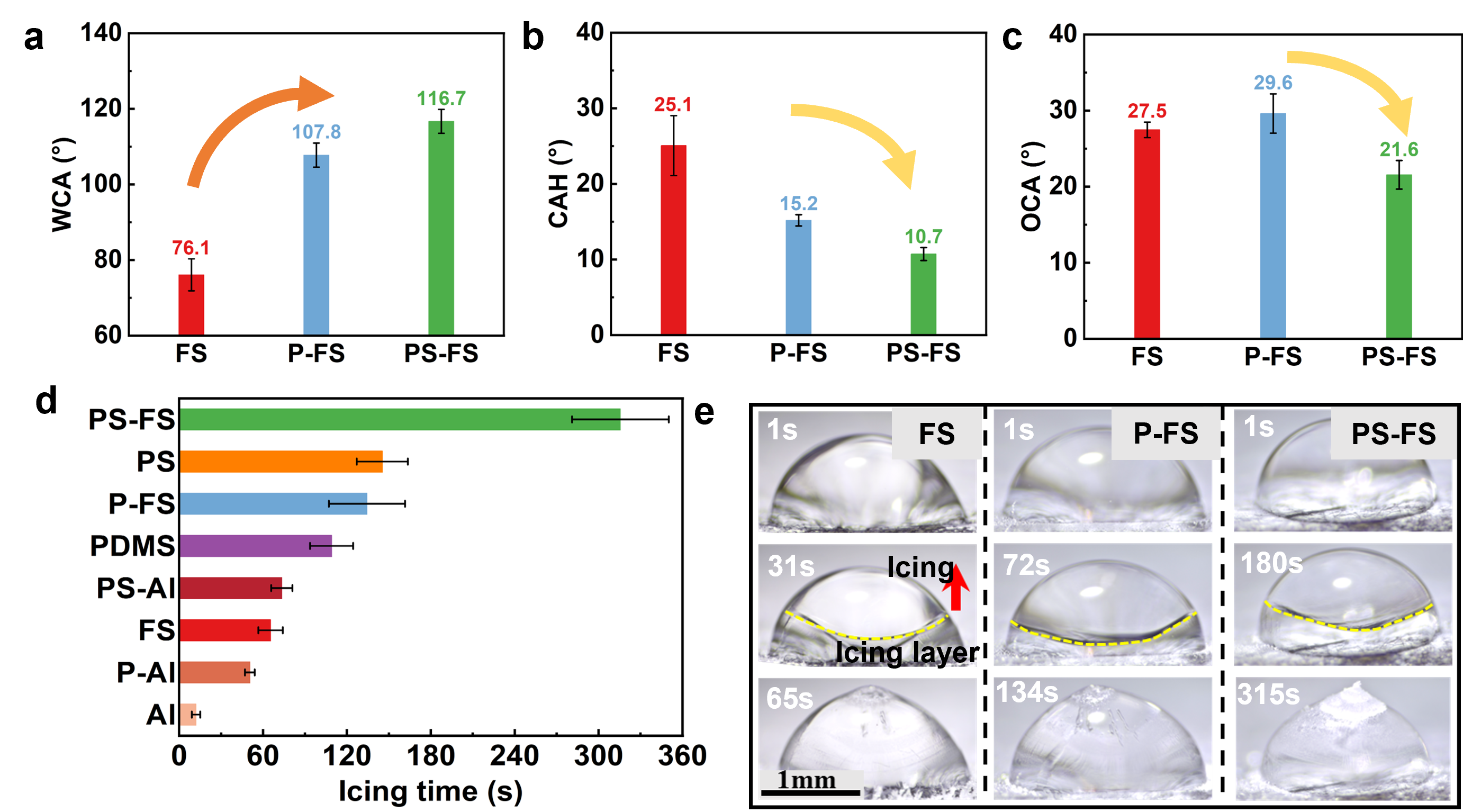


**Figure S5** Wettability of icephobic materials: a) WCA, b) CAH and c) OCA; d) freezing time of different materials at -20°C; e) icing process of FS, P-FS, and PS-FS.

The icing time was also related to the nucleation rate ($R(T)$) of ice crystals:[6]

$R(T)=R_{\text{bulk }}(T)V+R_{\lg}(T)S_{\lg}+R_{\mathrm{sl}}(T)S_{\mathrm{sl}}$ (S5)

where $R(T)$, $R_{\text{bulk }}(T)$, $R_{\lg}(T)$ and $R_{\mathrm{sl}}(T)$ are the total nucleation rate, volume nucleation rate, liquid-gas nucleation rate and solid-liquid interface nucleation rate dependent on temperature, respectively. *V*, $S_{\lg}$ and $S_{\mathrm{sl}}$ represent liquid volume, liquid-gas contact area and solid-liquid contact area individually. $T$ represents surface temperature. When surface hydrophobicity increased (from metal or fibre surfaces to icephobic polymer surfaces), the solid-liquid contact area decreased, slowing heat transfer and nucleation rate to delay the supercooling and freezing times of the droplets. When the solid-liquid contact area decreased, the nucleation rate of droplets at the solid-liquid interface decreased relative to the total nucleation rate,[6] promoting a more homogeneous nucleation process, thereby increasing the nucleation barrier and delaying the freezing time of the droplets.

The droplet icing process of FS, P-FS, and PS-FS is illustrated in **Figure S5e**. A white layer of ice was discernible at the droplet-solid interface (the yellow dashed line). This ice layer was formed at the solid-liquid interface and spread towards the top region of the droplet (the red arrow), ultimately resulting in complete freezing. Ice nuclei initiated at the interface and swiftly propagated upward. The interfacial resistance must be overcome to form an ice-water boundary before the growth of ice crystals. During the very early stage, the small volume of ice nuclei resulted in a large specific surface area, dominated by the interfacial process. The initiation of icing led to an increase in the system free energy, indicating the demand for additional energy input for a non-spontaneous process. However, once the ice nuclei exceeded a certain size, the specific surface area decreased, and the phase transformation process took over as the dominant mechanism. During this stage, freezing resulted in a decrease in free energy, becoming a spontaneous process, and the ice nuclei rapidly extended upward to complete the freezing process.[7]

The accumulation of frost on the prepared sample FS and PS-FS surfaces after 15 cycles of testing was illustrated in **Figure S6**. In low-temperature and high-humidity conditions, the porous FS adsorbed substantial quantities of water vapour, leading to frost formation from the edges to the centre. After 20 minutes, robust ice and a dense frost layer were formed on the FS surface. The surface of PS-FS was predominantly adorned with small droplets or ice crystals, forming a loosely structured frost layer at the edges. A large area of the PS-FS remained frost-free and rime frost was formed at the edge after 15 cycles. Compared to the rough surface of FS, the smooth PS-FS surface delayed the formation and growth of frost.


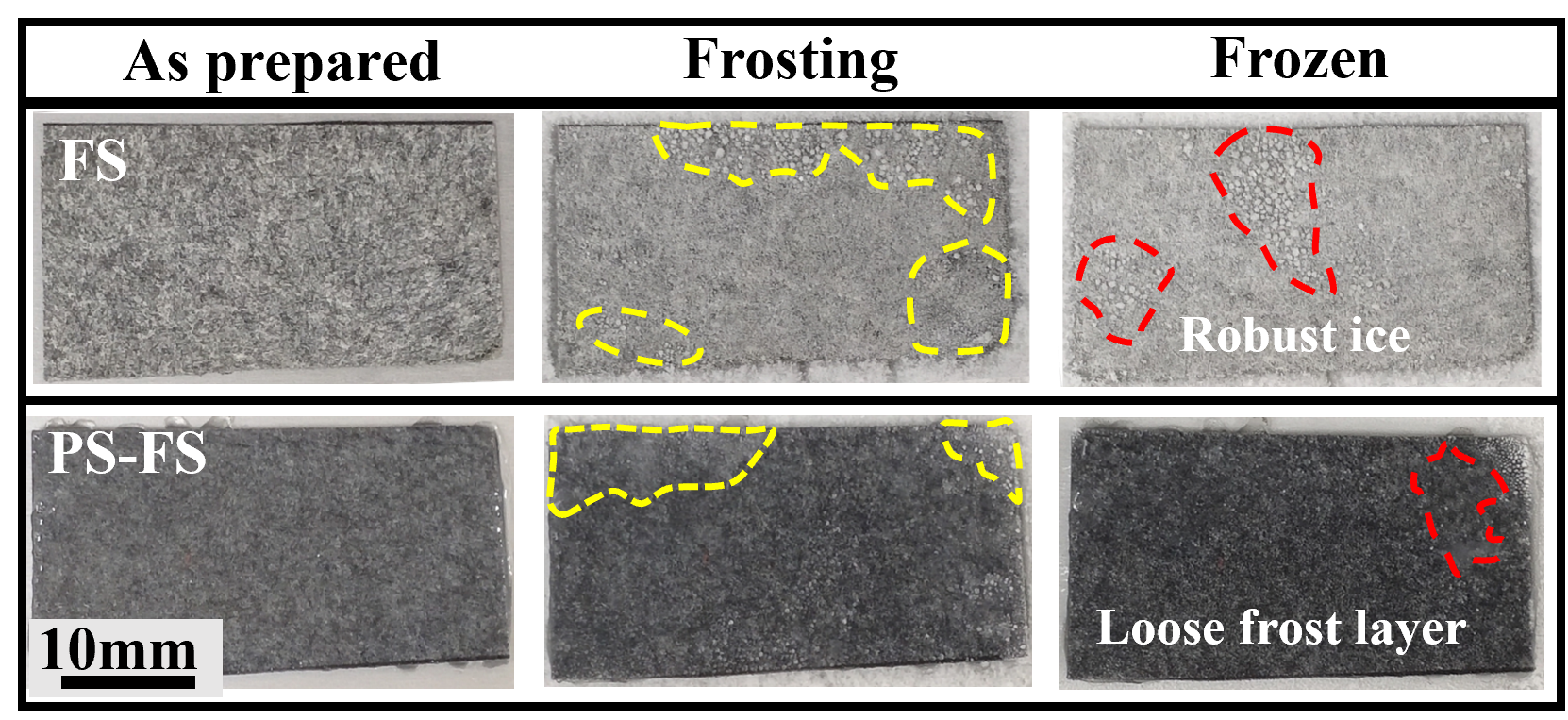


**Figure S6** Anti-frosting test results of FS and PS-FS samples

**Section S5: Supplementary of ice adhesion strength**

To compare the ice shear force-time curves at -10℃, the Y-axis was set as 0 to 32 N for each sample and summarised in **Figure S7a** to **S7h**. PS-FS and P-FS showed much lower detachment force than PS-Al and P-Al, which proved the advances of interfacial elastic instability in reducing ice adhesion strength.


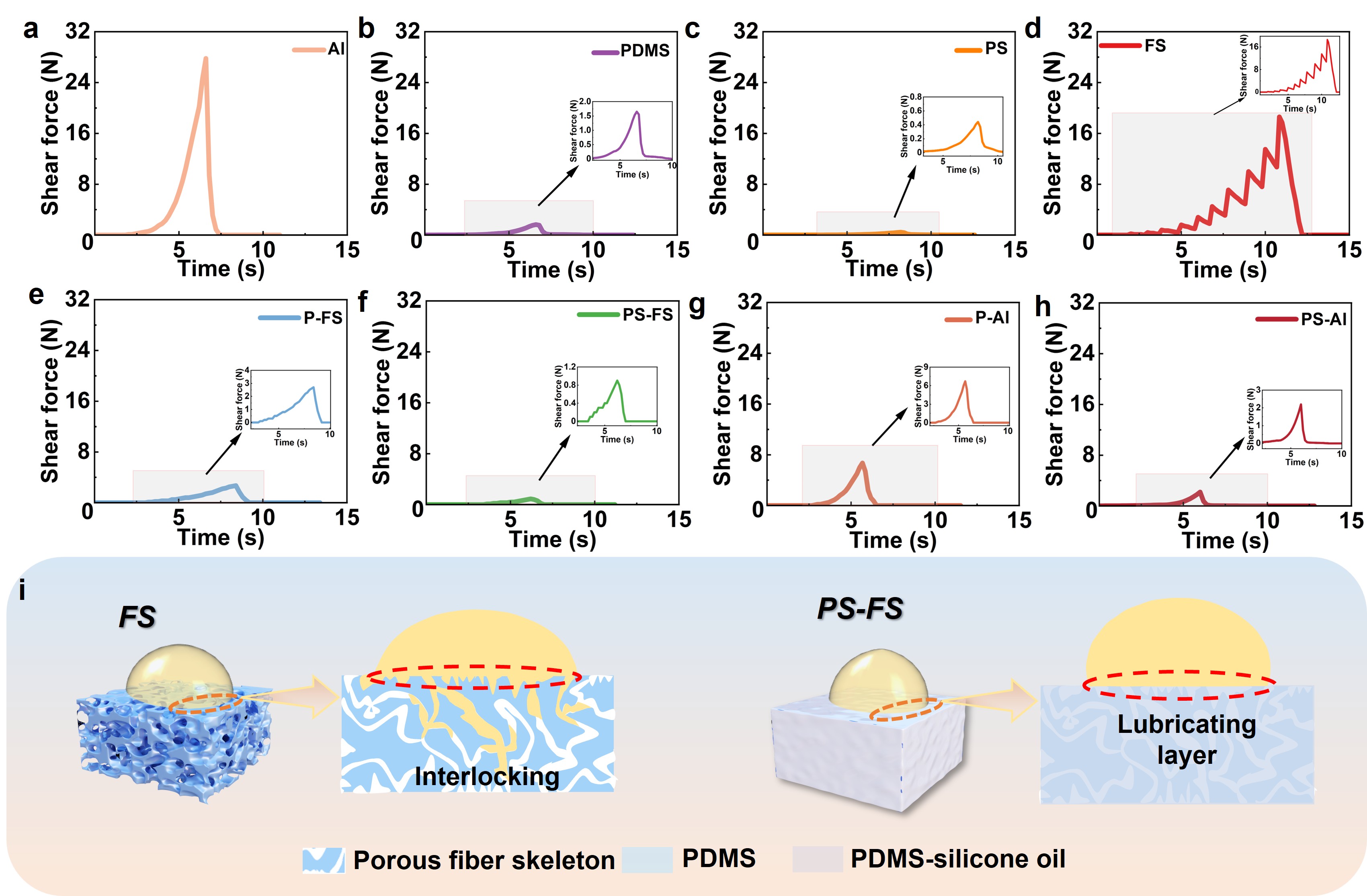


**Figure S7** a)-h) Comparison of the ice detached shear force-time curves of eight types of icephobic materials at -10℃; i) schematic diagram of the de-icing process and fracture surface of FS and PS-FS.

The mechanism of lowering ice adhesion strength by lubricating layer was schematically illustrated in **Figure S7i.** Due to the low freezing point of silicone oil, it remained in a liquid state at the experimental temperature, providing lubrication on the material surface. In our previous work, it was mentioned that when silicone oil was impregnated onto a rough surface, the initial ice formation occurred at the water-oil interface.[8] The presence of an oil layer on the surface could eliminate potential nucleation sites, transforming the solid-solid interface between the water droplet and the surface into the liquid-liquid interface. This transformation removed pinning points and reduced the solid-liquid contact area, inhibiting ice nucleation on the surface and contributing to an extended icing time. When the surface hydrophobicity was enhanced, it also reduced the solid-liquid contact area. It was beneficial for slowing down the heat transfer process and delaying droplet supercooling and freezing time. Additionally, a noticeable elastic modulus mismatch existed among polymers, fibres, and ice, leading to the change in stress distribution at the interface, intensifying stress concentration effects and increasing the likelihood of microcrack initiation at the solid-ice interface, further lowering ice adhesion strength.[9] Given the synergistic effect of high-strength fibre skeleton and lubricant, the multiple-phase icephobic material exhibited extremely low ice adhesion strength and optimal ice-repellent performance, demonstrating the advantage of the interfacial elastic instability.

The ice adhesion strength was also evaluated at lower temperatures (from -10℃ to -25℃), which were summarised in **Figure S8a** to **S8e**. For the flat Al surface, PDMS-coated Al, and P-FS, the ice adhesion strength increased linearly with the decrease of temperature. Regarding the FS, a steep increase of ice adhesion between -15℃ and -20℃. The ice adhesion strength was then kept around 330 kPa. The reason was that the decrease in temperature enhanced the mechanical interlocking between the ice and FS substrate. When the ice detached from the FS surface, a layer of FS film was also removed by the ice (as shown in Figure S8a and **S9g**). For PS-FS, due to the hybrid effect of the lubricant layer and interfacial elastic instability, the ice adhesion strength always maintained low values (lower than 10 kPa at -25℃) at different temperatures.


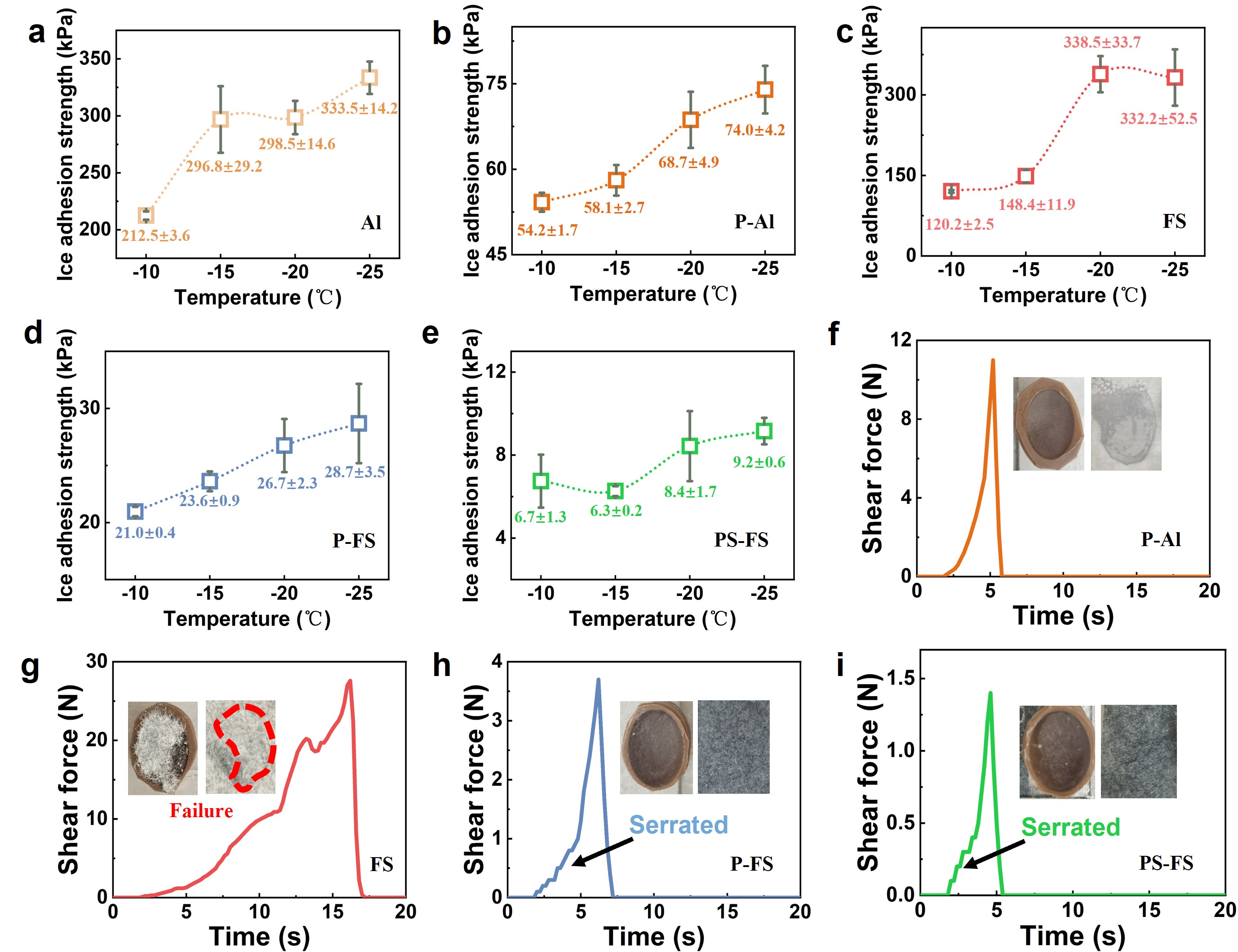


**Figure S****8** Ice adhesion strength of the samples at different temperatures: a) Al, b) P-Al, c) FS, d) P-FS, e) PS-FS; the curves of ice detached force of various samples at -25℃: f) P-Al, g) FS, h) P-FS, i) PS-FS

The curves of ice detached force of various samples at -25℃ were provided in **Figure S8f** to **S8i**. The ice detached force of the Al-based icephobic samples remained smooth with a flat ice fracture surface (Figure S8f). However, the ice detached shear force of the FS-based samples showed an odd variation (Figure S8g). Due to the porosity and hydrophilicity of the rough FS surface, the decrease of temperature enhanced the mechanical interlocking between the FC framework and ice, leading to some damage to the FC surface during the test once the shear force was higher than the strength of the FC inner layers. Both the FS fracture surface and the ice fracture surface remained rough, and fibre shedding was clearly observed on the ice bottom surface (Figure S8g). However, the P-FS and PS-FS samples still maintained the serrated load-time curves and the flat feature on the ice fracture surface, which proved the effectiveness of interfacial elastic instability at low temperatures.


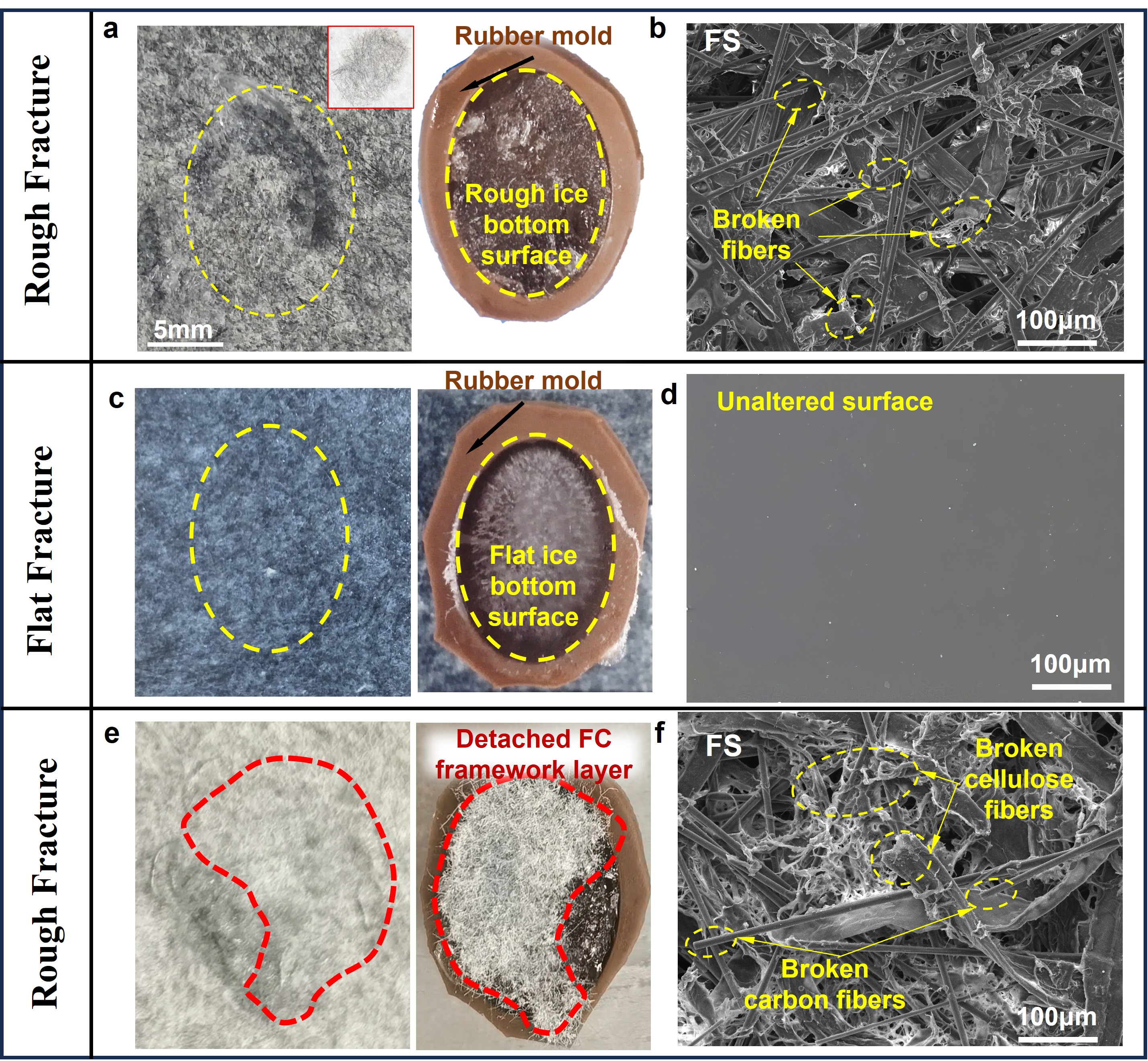


**Figure S9** a) The macro morphology and rough fracture surface of FS after ice adhesion test (-10℃); b) surface morphology of FS after ice adhesion strength test; c) the macro morphology and flat fracture surface of all other FS-based samples; d) surface morphology of all other FS-based samples after ice adhesion strength test; e) The macro morphology and rough fracture surface of FS after ice adhesion test (-25℃); f) surface morphology of FS after ice adhesion strength test (-25℃).

Figure S9 shows the enlarged fracture surface (both the sample surface and the ice bottom surface) of FS and other FS-based icephobic composites after the ice adhesion test. FS is a composite of fibre skeleton prepared by carbon fibre, aramid fibre, cellulose fibre and TPU resin binder. The surface of carbon fibres is chemically inert and has certain hydrophobicity. Aramid fibres contain polar amide bonds, and the high specific surface area increases their ability to spread water droplets. Cellulose fibres are highly hydrophilic because they contain a large amount of hydroxyl (-OH) and micropores, further promoting capillary water absorption. Since FS has high porosity and open pore structures, water droplets could penetrate the interior of the material after spreading on the surface. Under low-temperature conditions, the penetrated water was solidified and caused strong mechanical interlocking between ice and fibres. Due to the non-homogeneous microstructures, the detachment of ice was a gradual process along the interface. Then, the shear force starts to drop at around 3-4 N or even lower in Figure 4d. The detachment can happen at fibre/fibre, ice/fibre, and even ice/ice, which leads to a rough fracture surface and the shedding of fibres. With the decrease of temperature, the increase of interlocking and ice modulus induces a rougher fracture surface and more shedding (Figure S9a in SI).

**Section S6:** **Evaluation of durability**

Upon increasing silicone oil content, the ice adhesion strength on the surface of the icephobic materials decreased gradually. For the as-prepared sample, the surface was rich in oil film, resulting in lower ice adhesion strength. As the icing/de-icing cycle continued, the ice adhesion strength of the samples initially rose rapidly and then stabilised, which may be related to the amount of surface oil. It was hypothesised that if the silicone oil content increased to a supersaturated state. This supersaturation would prompt the presence of silicone oil on the surface, resulting in an exceptionally low initial ice adhesion strength. However, the thick oil layer was more susceptible to depletion. When the lubricant content was high, external forces (gravity, resistance, etc.) could remove the excessive surface lubricant, reaching a steady balance. Additionally, the existence of a “cloaking layer” led to the consumption of the lubricant during icing/deicing cycles,[10] thereby lowering the oil layer content on the surface. If no additional silicone oil was introduced to replenish the surface, the ice adhesion strength would escalate after several icing/de-icing cycles.[11]

**
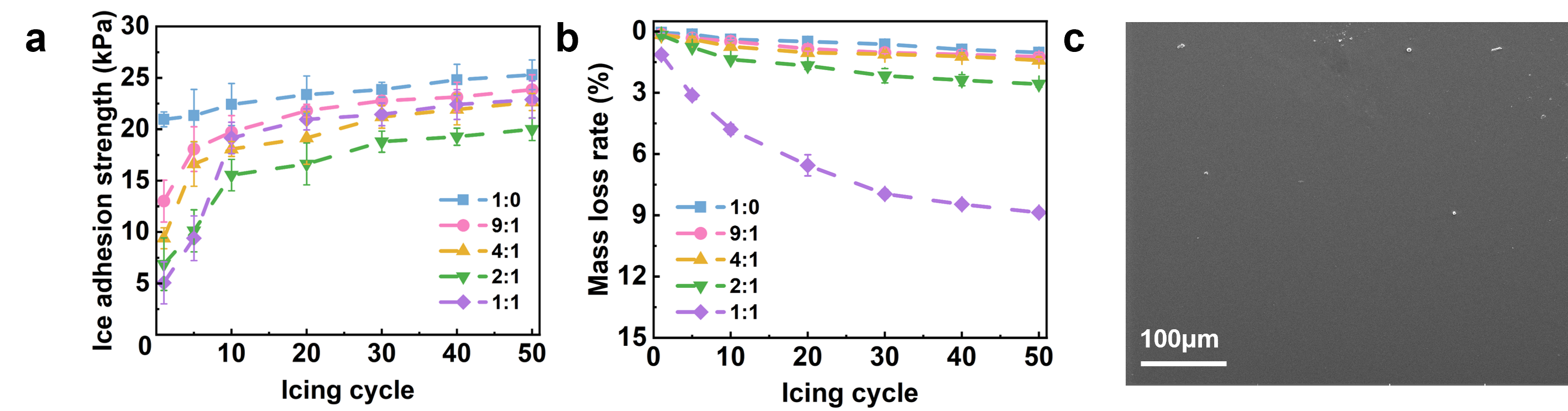
**

**Figure S10** a) ice adhesion strength of PDMS with different ratios of silicone oil during 50 icing/de-icing cycles; b) mass loss rate of PDMS with different ratios of silicone during 50 icing/de-icing cycles; c) SEM morphology of PS-FS after the 50_th_ icing/de-icing cycle


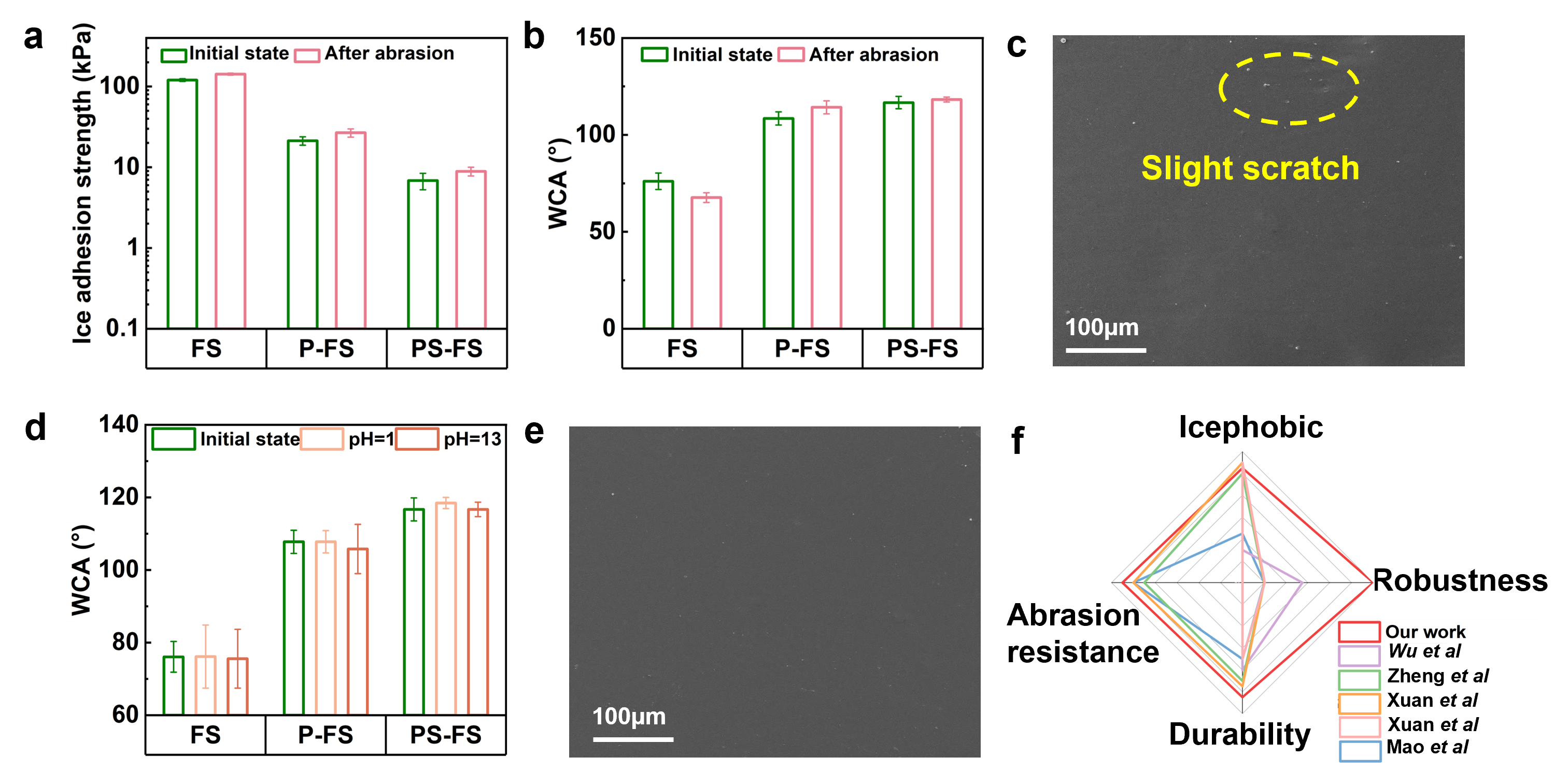


**Figure S11** a) comparison of ice adhesion strength after sandpaper abrasion; b) comparison of WCA after sandpaper abrasion; c) SEM morphology of PS-FS after sandpaper abrasion; d) comparison of WCA after chemical resistance; e) SEM morphology of PS-FS after soaking in acid/alkali solution; f) comprehensive comparison between the icephobic surfaces and some typical results from the literature.

**Figure S11a** presents the comparison of ice adhesion strength before and after the surface abrasion. The ice adhesion strength of the FS, P-FS, and PS-FS slightly increased to 142.4±4.0 kPa, 26.7±3.1 kPa, and 8.9±1.1 kPa, respectively. Both P-FS and PS-FS kept similar hydrophobicity and surface morphology after the abrasion with similar WCA values in **Figure S11b**. The surface morphology of PS-FS after abrasive paper friction merely showed small scratches as shown in **Figure S11c**. The synergistic effect of FC frameworks and lubricant oil layer enhanced good wear resistance. **Figure S11d** illustrates no significant variation in water contact angle after being subjected to HCl (pH=1) and KOH (pH=13) solutions. The surface morphology of the PS-FS sample also remained consistent after immersion in acid and alkali solutions, as shown in **Figure S11e**.

The comparison of comprehensive properties between this work and other reported icephobic surfaces/coatings/materials was summarised and discussed in **Figure S11f**. The icephobic surfaces, guided by the interfacial elastic instability principle in this study, showed much better comprehensive performance.[12] Besides achieving similar icephobic performance, long-term lifetime, and good abrasion resistance, the icephobic surfaces based on the interfacial elastic instability principle in this study also showed much improved mechanical robustness by promoting the tensile strength from less than 5 MPa to around 15 MPa. Moreover, in consideration of the wide usage of composite materials in the structural surfaces of aviation and wind turbines, the proposed interfacial elastic instability principle for developing advanced icephobic surfaces with multiple-phase FC frameworks exhibited great potential to integrate robust icephobic surfaces with the structural surfaces directly for convenient and long-lifetime service.

**Section S7: Supplementary finite element analysis**


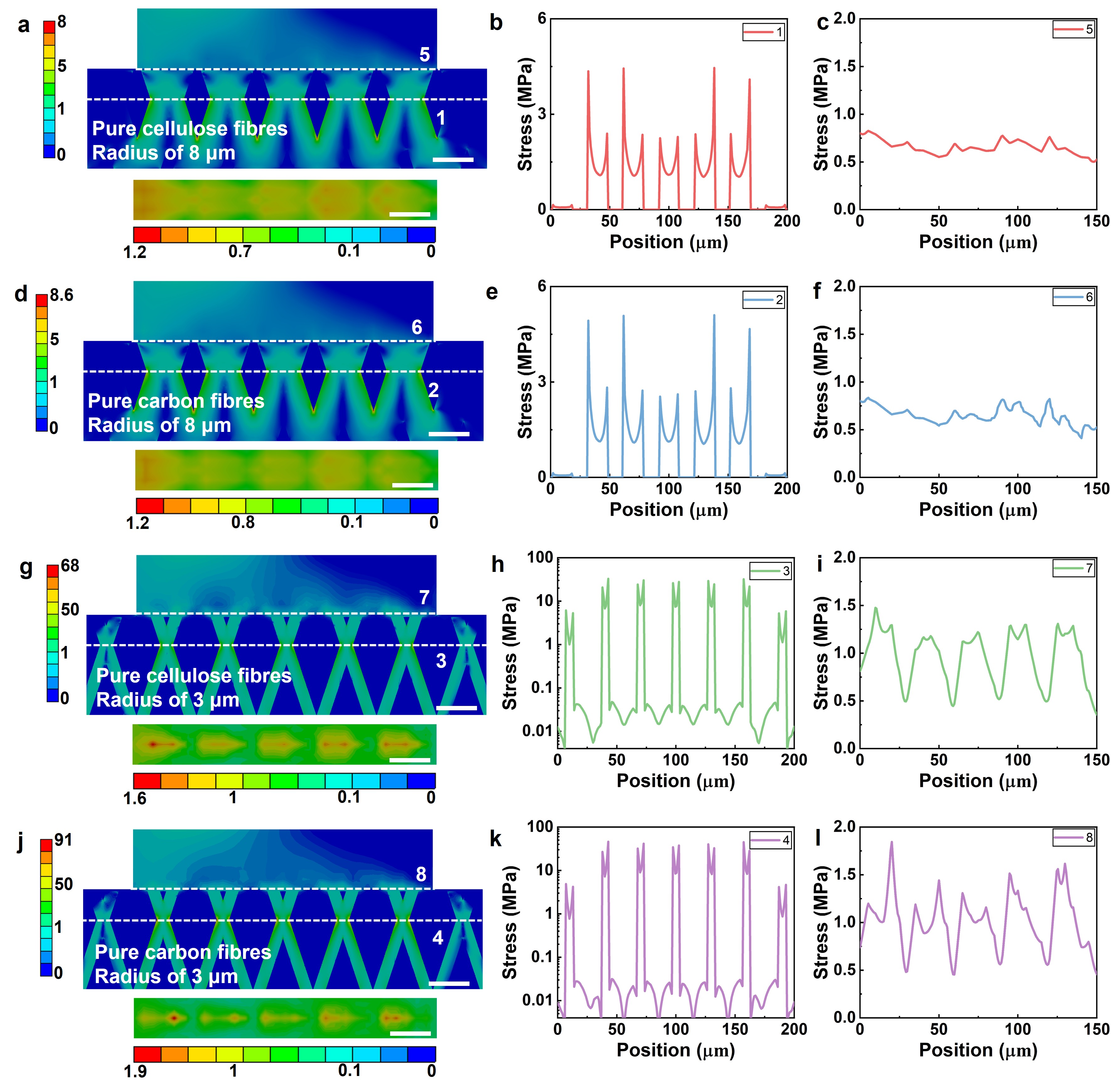


**Figure S12** Induced stress at the cross-sectional surface and ice bottom surface: a) pure cellulose fibre with a radius of 8 μm, d) pure carbon fibre with a radius of 8 μm, g) pure cellulose fibre with a radius of 3 μm, and j) pure carbon fibre with a radius of 3 μm; stress distribution along the select lines inside the icephobic materials: b) line 1 in Figure S12a, e) line 2 in Figure S12d, h) line 3 in Figure S12g, and k) line 4 in Figure S12j; stress distribution along the select lines on the ice bottom surfaces: c) line 5 in Figure S12a, f) line 6 in Figure S12d, i) line 7 in Figure S12g, and l) line 8 in Figure S12j; All the scale bars represented the length of 20 μm and the external shear loads (1MPa) were applied from the left side of the ice.

To further explore the influence of fibre modulus, size, and the porosity of the FC framework on interfacial elastic instability and stress undulation, another four simulation models were established. The key difference was the components of the FC frameworks, which consisted of pure cellulose fibres with a radius of 8 μm (**Figure S12a** to **S12c**), pure carbon fibres with a radius of 8 μm (**Figure S12d** to **S12f**), pure cellulose fibres with a radius of 3 μm (**Figure S12g** to **S12 i**), and pure carbon fibres with a radius of 3 μm (**Figure S12k** to **S12l**).

Through the comparison of Figure S12a, Figure S12d, Figure S12g, and Figure S12j, the increase of fibre modulus, the decrease of fibre thickness, and the rise of framework porosity were positive to prompt the interfacial elastic instability, further resulting in more undulatory interfacial stress at the ice/composites interfaces. For example, the stress on the ice bottom induced by the cellulose fibres with a radius of 3 μm in Figure S12i was in the range of 0.5 MPa to 1.5 MPa, which was lower than that induced by the carbon fibres with a radius of 3 μm in Figure S12l (ranging from 0.4 MPa to 1.9 MPa). The high modulus of carbon fibres could induce larger stress undulation than the low modulus cellulose fibre. On the other hand, when the modulus was kept constant, the stress undulation of the carbon fibres with a radius of 8 μm in Figure S12k was one order larger than that of the carbon fibres with a radius of 8 μm in Figure S12e. Also, the stress undulation of the ice bottom surface in Figure S12l was >1 MPa, higher than the value in Figure S12f (<0.4 MPa). The decrease of fibre radius and the rise of framework porosity increased the interfacial elastic instability and prompted the undulatory interfacial stress.

Notably, the above analysis merely focused on the efficiency in inducing interfacial elastic instability and ignored the demands of the mechanical properties. For the development of practical icephobic surfaces, the modulus, size of fibres and the porosity of FC frameworks need to be considered comprehensively.

**Section S8: Supplementary of Method**


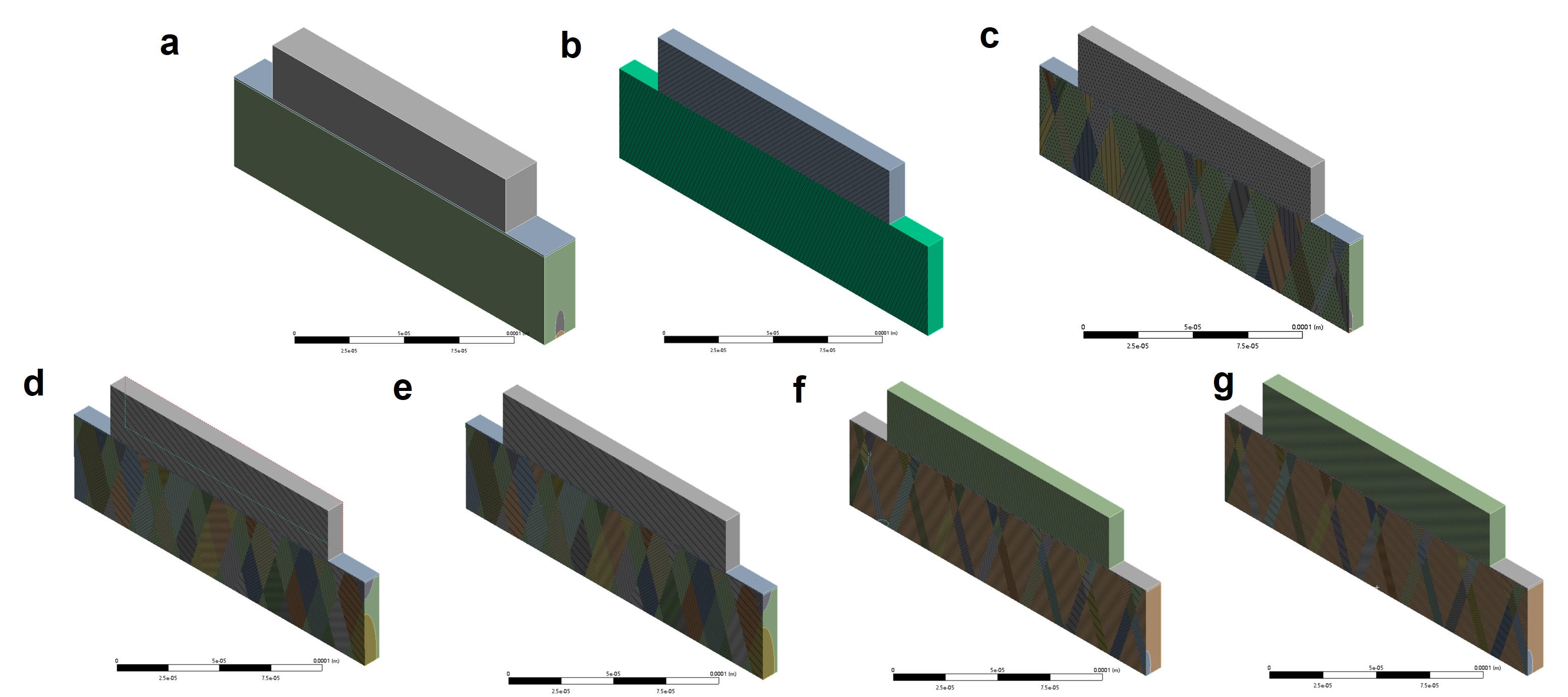


**Figure S13** a) schematic model design of numerical simulation in this study; the illustration of ice/solid section structure for different models: b) ice and PDMS coating; c) ice and the multiple-phase FC framework impregnated with PDMS; ice and the single-component FC framework impregnated with PDMS: d) cellulose fibres with a radius of 8 μm, e) carbon fibres with a radius of 8 μm, f) cellulose fibres with a radius of 3 μm, and g) carbon fibres with a radius of 3 μm.


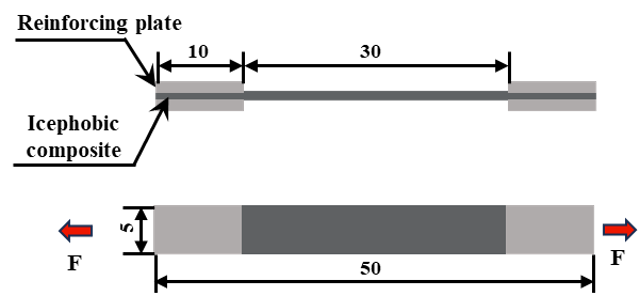


**Figure S14** Dimensional diagram for mechanical performance testing of multi-component icephobic materials


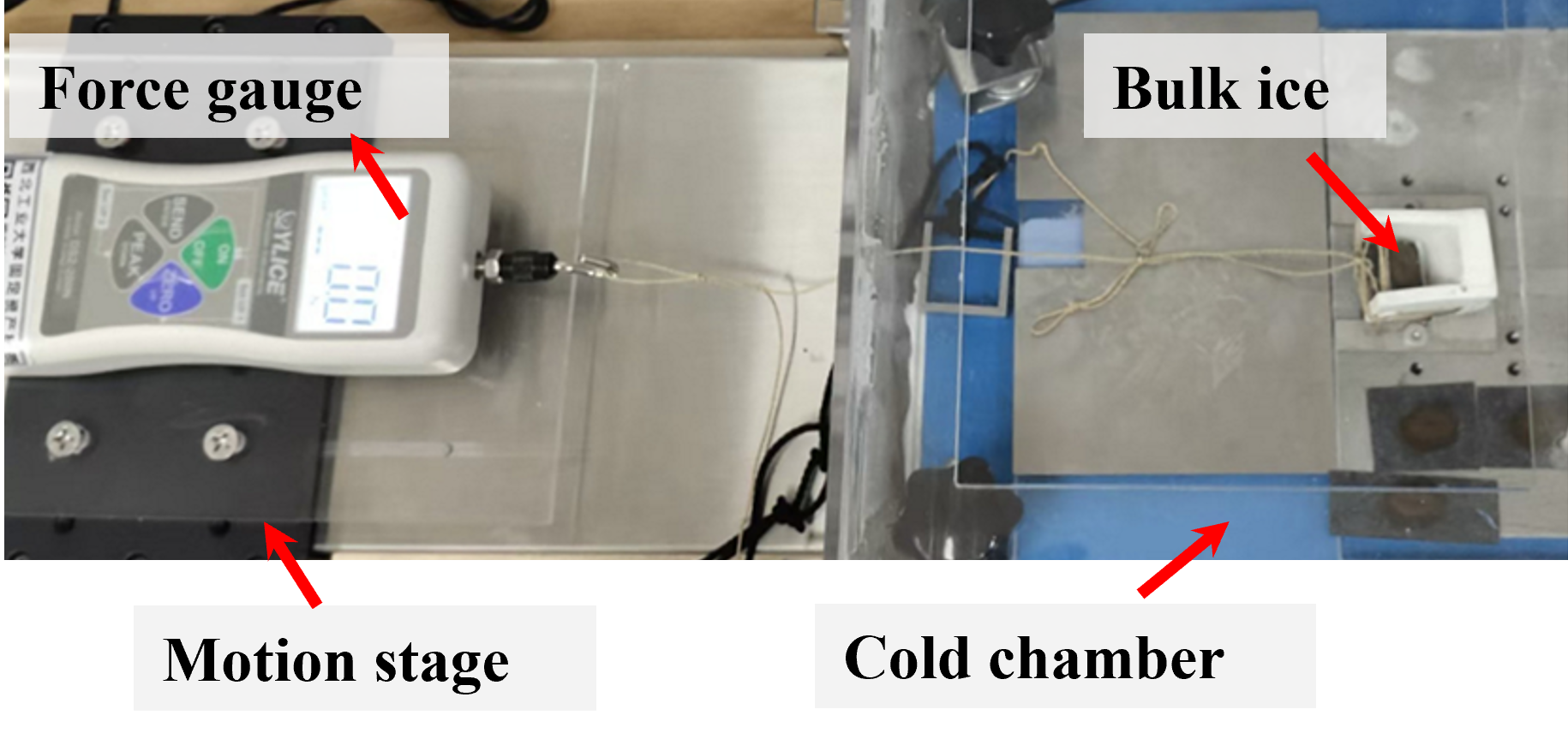


**Figure S15** Ice adhesion strength measuring equipment

The formula for calculating ice adhesion strength is as follows:

$$\tau=\frac{F}{A}$$

where $F$ refers to the shear force and $A$ is the icing area.


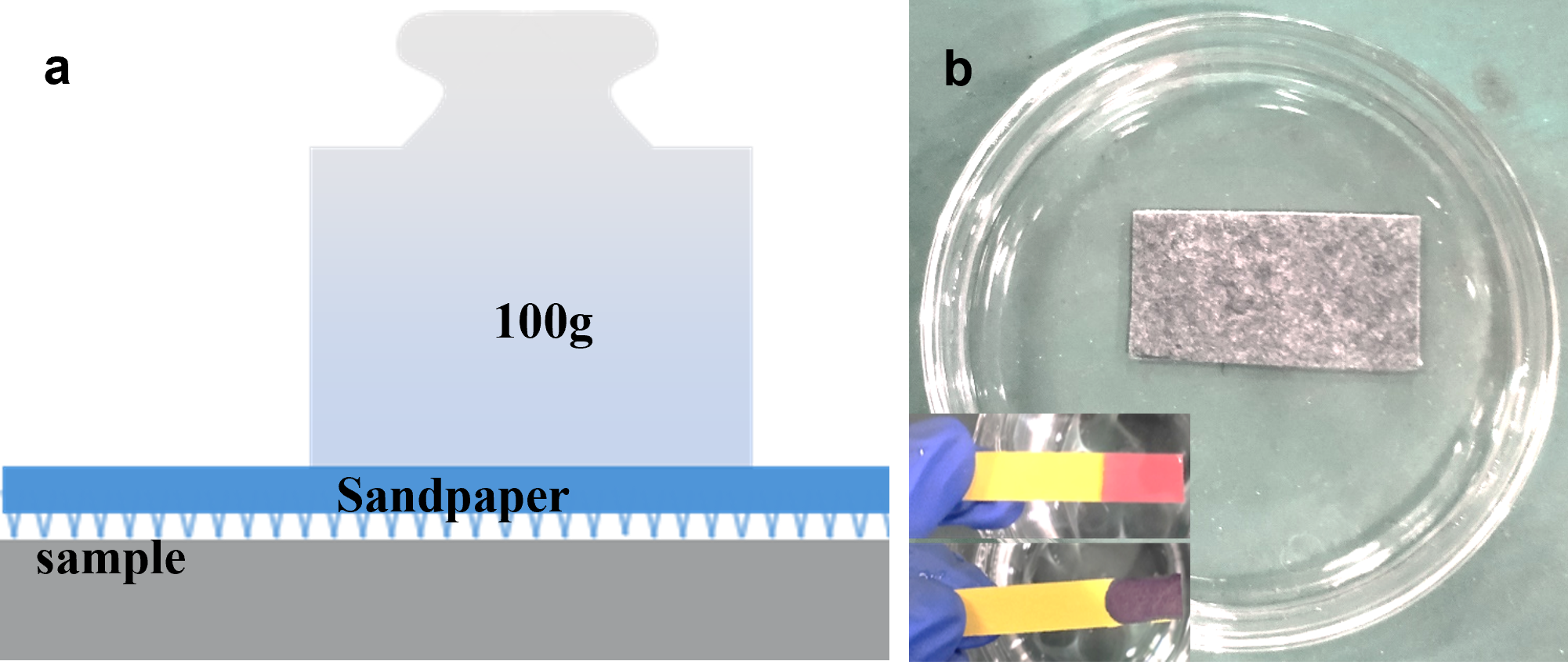


**Figure S16** a) schematic diagram of sandpaper abrasion; b) photograph of icephobic material for chemical resistance test

**References**

[1] M. K. Chaudhury, K. H. Kim, Shear-induced adhesive failure of a rigid slab in contact with a thin confined film, *Eur. Phys. J. E:Soft Matter Biol. Phys.* 2007, 23, 175.

[2] K. Liu, C. Yang, S. Zhang, et al., Multifunctional slippery polydimethylsiloxane/carbon nanotube composite strain sensor with excellent liquid repellence and anti-icing/deicing performance, *Polymers* 2022, 14, 409.

[3] S. Heydarian, R. Jafari, G. Momen, Recent progress in the anti-icing performance of slippery liquid-infused surfaces, *Prog. Org. Coat.* 2021, 151, 106096.

[4] a) N. H. Fletcher, Size effect in heterogeneous nucleation, *Chem. Phys.* 1958, 29, 572; b) M. Wu, J. Wang, S. Ling, R. Wheatley, X. Hou, Microporous metallic scaffolds supported liquid infused icephobic construction, *J. Colloid Interface Sci.* 2023, 634, 369.

[5] M. I. Jamil, A. Ali, F. Haq, Q. Zhang, X. Zhan, F. Chen, Icephobic strategies and materials with superwettability: Design principles and mechanism, *Langmuir* 2018, 34, 15425.

[6] S. Gao, L. Jiao, M. Yi, J. Jin, X.Wang, Mechanism and research progress of anti/de-icing using hydrophobic/superhydrophobic surfaces, *Acta Aerodynamica Sinica* 2021, 39, 151.

[7] a) Z. Zhang, X. Y. Liu, Control of ice nucleation: freezing and antifreeze strategies, *Chem. Soc. Rev.* 2018, 47, 7116; b) N. Maeda, Brief overview of ice nucleation, *Molecules* 2021, 26, 392.

[8] D. Yang, R. Bao, A. T. Clare, K.-S. Choi, X. Hou, Hydrophobically/oleophilically guarded powder metallurgical structures and liquid impregnation for ice mitigation, *Chem. Eng. J.* 2022, 446, 137115.

[9] a) Z. He, S. Xiao, H. Gao, J. He, Z. Zhang, Multiscale crack initiator promoted super-low ice adhesion surfaces, *Soft Matter* 2017, 13, 6562; b) P. Irajizad, A. Al-Bayati, B. Eslami, et al., Stress-localized durable icephobic surfaces, *Mater. Horiz.* 2019, 6, 758; c) M. Wu, J. Wang, S. Ling, R. Wheatley, X. Hou, Microporous metallic scaffolds supported liquid infused icephobic construction, *J Colloid Interface Sci* 2023, 634, 369.

[10] J. D. Smith, R. Dhiman, S. Anand, et al., Droplet mobility on lubricant-impregnated surfaces, *Soft Matter* 2013, 9, 1772.

[11] K. Wang, S. Wang, Y. Zhu, Effect of the porous electrode geometry on the freezing of supercooled water, *Journal of Power Sources* 2024, 602, 234310.

[12] a) Y. Wu, W. She, D. Shi, et al., An extremely chemical and mechanically durable siloxane bearing copolymer coating with self-crosslinkable and anti-icing properties, *Composites, Part B* 2020, 195, 108031; b) H. Zheng, Z. Lai, G. Liu, Ice-shedding endurance enhancement of lubricated polyurethane NP-GLIDE coating through dual cross-linking with covalent and hydrogen bonds, *Adv. Funct. Mater.* 2023, 34, 2312543; c) S. Xuan, H. Yin, G. Li, et al., Trifolium repens L.-Like periodic micronano structured superhydrophobic surface with ultralow ice adhesion for efficient anti-icing/deicing, *ACS Nano* 2023, 17, 21749; d) S. Xuan, L. Zhuo, G. Li, et al., Micro/nano hierarchical crater-like structure surface with mechanical durability and low-adhesion for anti-icing/deicing, *Small* 2024, 20, 2404979; e) M. Mao, J. Wei, B. Li, L. Li, X. Huang, J. Zhang, Scalable robust photothermal superhydrophobic coatings for efficient anti-icing and de-icing in simulated/real environments, *Nat. Commun.* 2024, 15, 9610.
